# Biodetoxification of Aflatoxin M1 in Artificially Contaminated Fermented Milk, Fermented Dairy Drink and Yogurt Using *Lactobacillus acidophilus* , *Lactobacillus plantarum* , *Lactobacillus reuteri* , and *Lactobacillus rhamnosus* and Its Effects on Physicochemical Properties

**DOI:** 10.1002/fsn3.70175

**Published:** 2025-04-24

**Authors:** Fatemeh Moradkhani, Seyed Saeed Sekhavatizadeh, Mohammad Hosein Marhamatizadeh, Maryam Ghasemi

**Affiliations:** ^1^ Faculty of Veterinary Medicine, Kazeroon Branch Islamic Azad University Kazerun Iran; ^2^ Department of Food Science and Technology Faculty of Fars Agricultural and Natural Resources Research and Education Center, AREEO Shiraz Fars Iran; ^3^ Department of Food Hygiene, Faculty of Veterinary Medicine, Kazerun Branch Islamic Azad University Kazerun Iran; ^4^ Department of Bacteriology, Faculty of Medicine, Kazerun Branch Islamic Azad University Kazerun Iran

**Keywords:** aflatoxin, biodegradation, fermented milk, lactic acid bacteria, probiotics

## Abstract

The aim of this research was to examine the occurrence of aflatoxin M1 (AFM1) in milk collected from Shiraz, Iran. Moreover, the capacity of the four probiotic species to reduce the concentration of AFM1 was assessed. For this research, 10 raw milk samples were collected randomly in Shiraz, Iran, in January 2023. The analyses were performed using an HPLC system equipped with a fluorescence detector. Then, AFM1 artificially contaminated probiotic drink milk (PDM) containing *Lactobacillus plantarum* (LP), *Lactobacillus reuteri* (LRE), *Lactobacillus rhamnosus* (LR) (LABs) alone or in combination, fermented dairy drink containing *Lactobacillus acidophilus* (FDD‐LA), and yogurt were produced. The pH, total viable number of probiotics (TVNP), and rheology of the samples were measured. In all milk samples, the concentrations of AFM1 did not exceed the maximum threshold established by Iranian national standards. In the artificially spiked samples, the maximal reduction in AFM1 was noted in PDM_LP_ (96.5%), PDM_LP‐LRE‐LR_ (95.3%), yogurt (95.2%), and PDM_LR_ (93.8%). FDD‐LA revealed the lowest reduction in AFM1 (75.9%). The TVNP increased during processing, while the pH decreased in all the samples. AFM1 did not have any negative effect on TVNP or pH. The SEM images revealed that the AFM1‐treated samples had a less compact structure and more cavities. Similarly, the viscosity of the control sample without AFM1 was greater than that of the AFM1‐supplemented samples. The results showed that LP, LRE, LR, LA, and yogurt starter cultures have a strong ability to decrease AFM1 in milk. However, their effect on the textural properties of the product is not favorable.

## Graphical abstract

LABs have a strong ability to decrease AFM1 in milk. The maximal reduction in AFM1 was related to *Lactobacillus plantarum* . AFM1 did not have any negative effect on the total viable number of probiotics. The AFM1‐treated samples had a less compact structure and more cavities.

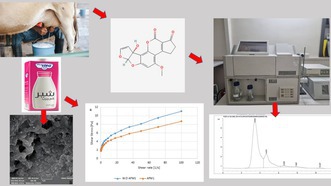

## 1 Introduction

Milk and dairy products serve as significant sources of calcium, protein, and other micronutrients. Milk and dairy products contribute nearly 50% of overall saturated fat consumption. Dairy products are a heterogeneous group of products that serve dual purposes as both food and raw materials in the production of various dairy products, including yogurt and fermented milk, butter, cream, and cheese (Holven and Sonestedt 2024). FDD‐LA is another type of dairy product that includes a diverse group of beverages, such as milk/juice drinks and yogurt beverages. A common characteristic of these products is their low pH and low viscosity (Janhøj et al. 2008).

AFM1 is a secondary metabolite derived from aflatoxin B1 (AFB1), a mycotoxin synthesized by *Aspergillus parasiticus* and *A. flavus* , that contaminates animal feed in warm regions globally. When animals ingest AFB1 present in these foods, they secrete AFM1 into their milk. A recent study revealed that exposure to AFM1 is linked to an increased risk of cancer (Wu et al. 2024).

AFM1 has the potential to influence kidney, liver, and immunological functions, thereby affecting the health of both animals and humans and contributing to liver damage. The EU standard limit for milk products is 50 ng/kg, while it is 25 ng/kg for infant milk products (European Commission 2023). The standard technique employed for measuring AFM1 levels in milk samples involves HPLC coupled with a fluorescence detector (Aghebatbinyeganeh et al. 2024).

In one study, (Iha et al. 2013) found that the presence of AFM_1_ was identified in 83% of the milk samples (> 3 ng/kg), with concentrations exceeding 20–760 ng/kg in powdered milk. The levels of AFM_1_ in fluid milk varied significantly (8–437 ng/kg). Both processing and storage conditions had minimal effects on the AFM1 concentration in milk and dairy products. The total concentration of AFM1 in cheese decreased by 3.2%, and by 6% in yogurt at a pH of 4.4. In contrast, compared with unprocessed milk, the average AFM1 concentration in curds was found to be 1.9 times greater, whereas whey showed a 0.6‐fold decrease. The prevalence and occurrence of AFM1 contamination in both milk and dairy products are influenced by the geographical origin of the products (Iha et al. 2013). Numerous studies have documented the prevalence of AFM1 contamination in milk and dairy products, including findings from Iran (Behtarin and Movassaghghazani 2024), Kosovo (Camaj Ibrahimi et al. 2024), Sri Lanka (Mudannayake et al. 2024), Egypt (Shahata and Wafy 2024), Ghana (Kortei et al. 2024), Serbia (Kos et al. 2024), and Nepal (Kafle et al. 2024). Controlling AFM1 is essential, given the significant risks it poses to human health and economic stability in all societies worldwide. Various methods have been developed for AFM1 detoxification, including both chemical and physical AFM1 detoxification techniques have been established to inactivate or remove it. Currently, consumers increasingly prefer products with the highest nutrient value and minimal changes in composition (Mousavi Khaneghah et al. 2017).

AFM1 is characterized by its stability during heat stress, which remains unaffected by typical domestic techniques such as pasteurization. Nevertheless, there is ongoing debate regarding its stability under pasteurization conditions. Research has suggested that pasteurization does not alter the level of AFM1, whereas other reports have shown a significant reduction (90%), potentially attributed to the breakdown of casein that occurs during heat conditions (Kafle et al. 2024).

Among the various biological techniques available, the detoxification of AFM1 using probiotic bacteria is considered the most advantageous. Additionally, LABs are acknowledged as effective microbial agents, generally recognized as safe (GRAS), and offer associated health benefits. The extensive application of these products in the food industry can be attributed to several factors, including their ability to produce organic acids, enzymes, vitamins, generate low‐calorie sweeteners, and ferment milk (cheese, buttermilk, curd, yogurt, etc.) (Patel 2024).

*Lactobacilli* are added to milk and dairy products to reduce AFM1 levels. For example, Erfanpoor et al. (2024) found that LP and *Lactobacillus brevis* isolated from Siahmazgi cheese can effectively reduce AFM1. The AFM1 levels decreased by 48%, 35.08%, and 39.81% in *L. brevis* , LP, and *L. brevis* + LP combination treatment, respectively. The range of reduction of AFM1 in milk by *Lactobacilli* isolated from Siahmazgi cheese ranged from 35.08%–49.01% (Erfanpoor et al. 2024). In the study conducted by Rezasoltani et al. (2022), the detoxification effects of the probiotics *Saccharomyces boulardii*, *Lactobacillus casei* , and *Lactobacillus acidophilus* on AFM1 in reconstituted milk were investigated. The results indicated that the highest AFM1 removal rate was achieved with a concentration of *S. boulardii* at 10^9^ cfu/mL for 90 min at 37°C, with a toxin concentration of 0.75 ng/mL, yielding a removal rate of 96.88 ± 3.79 (Rezasoltani et al. 2022).

Liu et al. (2020) emphasized that the biological decontamination of aflatoxins is particularly effective through the use of lactic acid bacteria (Liu et al. 2020). Demir and Tutun (2024) and Salem‐Bekhit et al. (2023) evaluated the detoxification of AFM1 in milk using *L. rhamnosus* and *Saccharomyces cerevisiae* . In their study, optimal conditions were determined, achieving a remarkable 98.4% detoxification of AFM1. This impressive result highlights the impact of probiotics on milk contamination (Salem‐Bekhit et al. 2023 and Demir and Tutun 2024). Among *Lactobacilli*, LP was the most effective strain, binding AFM1 at the highest rate and reducing AFM1 levels throughout the storage period of the yogurts. These findings support the significant role of probiotics in the detoxification of AFM1 in food products (Elsanhoty et al. 2014).

The organic acid produced by LAB contributes to biotransformation under specific conditions and can detoxify harmful compounds by deionizing them. In certain instances, the cell wall components of LAB, including proteins, peptidoglycans, and polysaccharides, play crucial roles in decreasing the availability of heavy metals and mycotoxins through mechanisms such as adsorption via ion exchange mechanisms (Patel 2017).

Following the consumption of contaminated milk, AFM1 undergoes conversion into carcinogenic metabolites within liver tissue, facilitated by P450 monooxygenase. This transformation is a result of demethylation, oxidation, and hydroxylation reactions. The epoxide form of AFM1 interacts with guanine residues in DNA, leading to mutations, a process that ultimately contributes to the development of liver cancer. The lactone and furan ring structures represent two essential sites for the toxicity of AFM1 (Burak and Samankova 2024).

The structural integrity of these two rings is crucial, as any cleavage can result in the detoxification of AFM1. Research indicates that probiotics can detoxify AFM1 in two distinct ways. First, AFM1 can be adsorbed by the wall carbohydrates of probiotics, thereby increasing its bioavailability. Additionally, the metabolites released by probiotics may facilitate the degradation of AFM1. Furthermore, AFM1 can bind to cell wall polysaccharides and peptidoglycans through noncovalent bonds, including hydrogen interactions and electrostatic and van der Waals interactions (Minuye 2021).

Moreover, the amount of AFM1 can be reduced by postbiotics. Postbiotics include cell wall‐derived substances (proteins, peptidoglycan, polar lipids, glycolipids, peptidoglycan, teichoic acids and exopolysaccharides) and cell‐free supernatants (peptides, fatty acids, proteins, vitamins, enzymes, and organic acids).

Postbiotic compounds demonstrate a wide range of diversity, influenced by the specific types of bacteria and the culture media involved. However, there is insufficient evidence suggesting that different compounds may interact with AFM1. It is possible that postbiotic compounds, especially those with exopolysaccharides, can interact with AFM1, helping to prevent its absorption and potentially reducing toxicity levels. LABs are the predominant probiotics utilized to reduce AFM1 (Burak and Samankova 2024).

The addition of prebiotics with probiotics leads to an increase in AFM1 binding. Studies have shown that the ability of probiotics to bind to AFM1 is affected by various factors, such as the type of bacterial strain used, the initial concentrations of prebiotics, probiotics, and AFM1, and the temperature and duration of incubation. As a result, prebiotics can be proposed as a biological tool for decreasing AFM1 concentrations (Macit et al. 2024).

*Lactobacillus* spp. has demonstrated potential for reducing AFM1 levels in milk and dairy products, increasing these levels from less than 10% to as high as 99%. Additionally, the effect of *Lactobacillus* spp. on AFM1 residues is influenced by various factors, including the concentration of aflatoxin, type and viability of bacteria, bacterial population, incubation and storage time, and fermentation conditions (Zareie et al. 2024).

*Lactobacilli* are added to milk and dairy products to reduce AFM1 levels. For example, Erfanpoor et al. (2024) found that LP and *Lactobacillus brevis* isolated from Siahmazgi cheese can reduce AFM1 levels. AFM1 levels decreased by 48%, 35.08%, and 39.81% in *L. brevis* , LP, and *L. brevis* + LP combination treatment, respectively. The reduction of AFM1 in milk by *Lactobacilli* isolated from Siahmazgi cheese ranged from 35.08%–49.01% (Erfanpoor et al. 2024). In a study conducted by Rezasoltani et al. (2022), the detoxification effects of the probiotics *Saccharomyces boulardii*, *Lactobacillus casei* , and *Lactobacillus acidophilus* on AFM1 in reconstituted milk were investigated. The results indicated that the highest AFM1 removal rate was achieved with a concentration of *S. boulardii* at 10^9^ CFU/mL for 90 min at 37°C with a toxin concentration of 0.75 ng/mL, yielding a removal rate of 96.88% ± 3.79% (Rezasoltani et al. 2022).

Liu et al. (2020) emphasized in their study that the biological decontamination of aflatoxins is particularly achieved through the use of lactic acid bacteria (Liu et al. 2020). Salem‐Bekhit et al. (2023) evaluated the detoxification of AFM1 in milk using *L. rhamnosus* and *Saccharomyces cerevisiae* . In Salem‐Bekhit et al. (2023) study, optimal conditions were determined, achieving a remarkable 98.4% detoxification of AFM1. This impressive result highlights the impact of probiotics on milk contamination (Salem‐Bekhit et al. 2023). Among *Lactobacilli*, LP was found to be the most effective strain, binding AFM1 at the highest rate and reducing AFM1 levels throughout the storage period of the yogurts. These findings support the significant role of probiotics in the detoxification of AFM1 in food products (Elsanhoty et al. 2014).

We believe that using LABs and yogurt starter cultures might help reduce AFM1 levels in dairy products. This study aims to demonstrate that incorporating probiotic dairy products can significantly decrease aflatoxin M1 levels, lessen its negative impact on health, and ultimately support public well‐being.

This research aimed to explore the presence of aflatoxin M1 in raw milk from Shiraz, Fars Province, Iran. It was conducted to identify a contaminated sample for subsequent experiments targeting aflatoxin mitigation. Aflatoxin M1 binds to the casein fraction, which results in a higher level of contamination in milk products than in raw milk. Therefore, the reduction in artificially spiked aflatoxin M1 in probiotic drink milk, fermented dairy drink, and yogurt in the presence of *Lactobacillus acidophilus* , *Lactobacillus plantarum* , *Lactobacillus reuteri* , *Lactobacillus rhamnosus* , and their combinations was assessed. We chose these *Lactobacillus* strains because they are “probiotic powerhouses” with proven efficacy. For example, *L. rhamnosus* and *L. plantarum* act like molecular sponges for aflatoxins (Trinder et al. 2016), while *L. acidophilus* thrives in harsh gut conditions. Additionally, all strains are safe for food use, making our results potentially significant for safer milk production practices (el Khoury et al. 2011; Tajik and Sayadi 2022).

## 2 Materials and Methods

### 2.1 Materials

UHT milk with the following characteristics was selected: 1.5% fat content, 16° Dornic acidity, 8% solids‐not‐fat (SNF), a freezing point of −0.565°C, and a density of 1.030 g/cm^3^. No bacterial growth was observed following culture and incubation at 37°C and 45°C. Low‐fat UHT cow milk was purchased from Pegah Dairy Co., Shiraz, Fars, Iran. No thermal processing was applied to kill microorganisms because it was sterilized under UHT conditions in the factory. However, the samples were heated at 90°C for 15 min based on production procedures, except for the fermented dairy drink. Freeze‐dried LA ATCC 4356, LR ATCC 53103, LP ATCC 1024, and LRE ATCC 23272 were used in this research. All strains were obtained from Persian type culture collection Karaj, Tehran, Iran, and Pardis Roshd Mehregan Co., Shiraz, Fars Province, Iran. All probiotics have human origin with nonpathogenic characteristics. The CH1 yogurt starter culture was obtained from Chr. Hansen (Denmark).

### 2.2 Sampling

Our objective was to identify aflatoxin‐contaminated milk samples to perform detoxification protocols on those specific batches. For this purpose, milk samples were collected across 3 days to ensure consistency and representation. By combining daily samples from each farm, researchers created composite samples that captured potential aflatoxin variability. Strict storage conditions at 4°C during collection and −20°C during transport preserved sample integrity, while light‐protected, sterile, sealed containers prevented aflatoxin degradation. This methodological rigor ensured reliable data for assessing probiotic efficacy in aflatoxin reduction (Aghebatbinyeganeh et al. 2024).

### 2.3 Extraction of AFM1 From PDM, C, FDD‐LA and Yogurt

A mixture of 5 mL of sample was combined with 41 mL of distilled water and then centrifuged at 5500 rpm for 4 min at 4°C. The fat was isolated, and the blue portion was extracted. The sample was filtered through a 0.45 μm Millipore cellulose acetate filter (Merck, Germany). The process involved the application of 100 mL of filtered liquid, placed on an immunoaffinity column with Aokin ImmunoClean C AFLA M1 (Aokin, Berlin, Germany) specific antibodies. Following the completion of the absorption phase, the filter was rinsed with distilled water prior to being removed and concentrated with acetonitrile. Subsequently, 100 μL of the sample was injected into a Hewlett‐Packard (Palo Alto, CA) model 1090 high‐performance liquid chromatograph (HPLC), with fluorescence detection, which was used to determine retention times and the fluorescence absorption spectra of AFM1 (Behtarin and Movassaghghazani 2024; ISIRI 2013).

### 2.4 HPLC Analysis

The measurement of AFM1 was conducted via the HPLC technique (ISIRI 2013). Aokin Immuno Clean AFLA M1 (Aokin, Berlin, Germany) was used as the AFM1 standard. For quantitative analysis, a 100 μL aliquot was injected into the HPLC system. Optimal separation was obtained with a C18 column (25 × 4.6 mm, 5 μm) at 40°C. At a pressure of 2400 psi, the mobile phase consisted of water/acetonitrile/methanol (6:2:2 v/v). The rinse rate was set at 1.1 mL/min. The fluorescence detector was operated at an excitation wavelength of 360 nm and an output wavelength of 440 nm. After each sample was injected, the area under the peak of the graph at the point of inhibition was measured, and this value was compared to the standard curve to assess toxin content. The recorded retention time was 10 min. To identify the AFM1 peaks in the sample chromatogram, the retention times were compared with those of the AFM1 standards analyzed under the same conditions. The peak area of the sample chromatogram was used, and the equation of the calibration curve was calculated to quantify AFM1. Signal‐to‐noise ratios of 3:1 and 10:1 were used for calculating the limit of quantification (LOQ) and the limit of detection (LOD), respectively.

**FIGURE 1:**
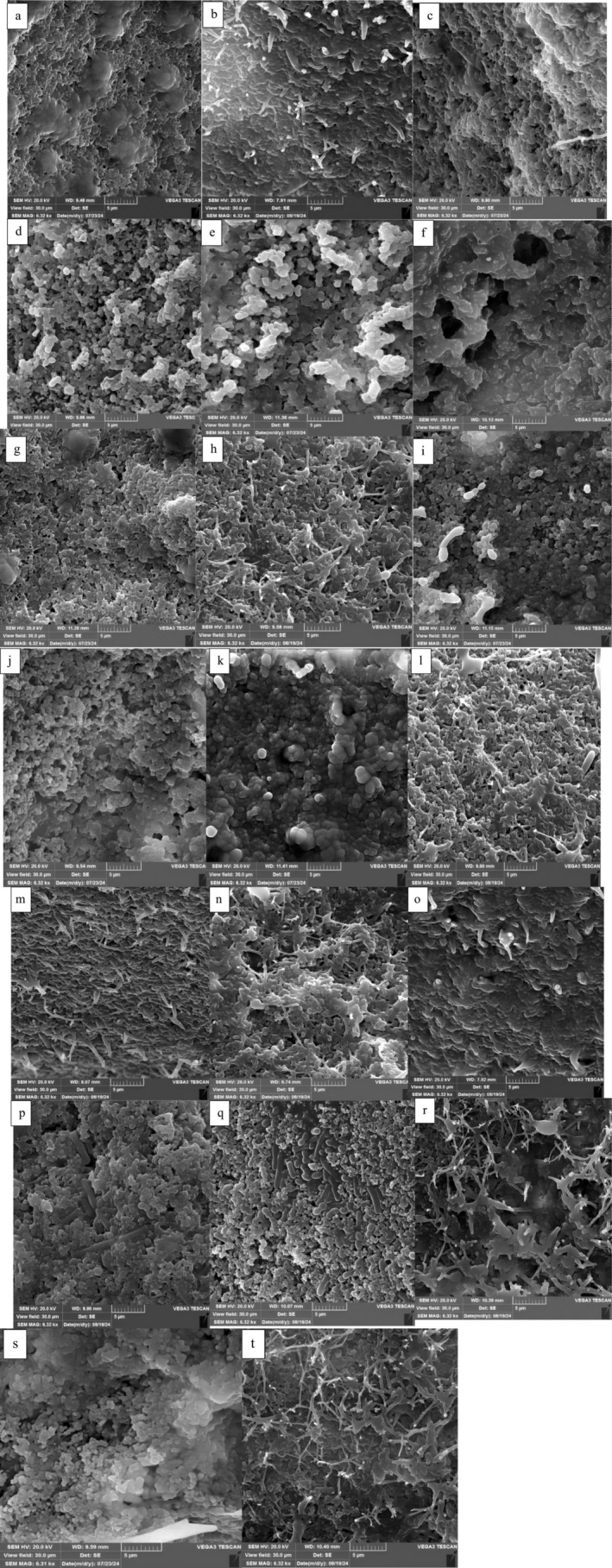
The SEM images of PDM_LP_ (a), PDM_LP_ with AFM1 (b), PDM_LRE_ (c), PDM_LRE_ with AFM1 (d), PDM_LR_ (e), PDM_LR_ with AFM1 (f), PDM_LP‐LRE_ (g), PDM_LP‐LRE_ with AFM1 (h), PDM_LRE‐LR_ (i), PDM_LRE‐LR_ with AFM1 (j), PDM_LR‐LP_ (k), PDM_LR‐LP_ with AFM1 (l), PDM_LRE‐LR‐LP_ (m), PDM_LRE‐LR‐LP_ with AFM1 (n), Yogurt (o), Yogurt with AFM1 (p), AM (q), AM with AFM1 (r), C (s), C with AFM1 (t) after storage period. AFM1, aflatoxin M1; FDD‐LA, fermented dairy drink containing *Lactobacillus acidophilus* ; FDD‐LA, fermented dairy drink containing *Lactobacillus acidophilus*; LA, *Lactobacillus acidophilus* ; LP, *Lactobacillus plantarum* ; LR, *Lactobacillus rhamnosus* ; LRE, *Lactobacillus reuteri* ; PDM, probiotic drink milk; PDM_LP_, PDM contains LP; PDM_LP‐LRE_, PDM contains LP and LRE; PDM_LR_, PDM contains LR; PDM_LRE_, PDM contains LRE; PDM_LRE‐LR_, PDM contains LRE and LR; PDM_LRE‐LR‐LP_, PDM contains LRE, LR and LP; PDM_LR‐LP_, PDM contains LRE and LP; Yogurt, contains starter culture.

Linearity was confirmed in all chromatographic analyses by evaluating the coefficient of determination (*r* ^2^) in the residual plots of the calibration curves (Figure 1s) (ISIRI 2013).

### 2.5 Preparation of Bacteria

Freeze‐dried LA ATCC 4356, LR ATCC 53103, LP ATCC 1024, and LRE ATCC 23272 were cultivated separately in MRS broth under anaerobic conditions at 30°C for 48 h. The LABs were subsequently centrifuged at 4000 rpm at 4°C for 15 min and washed twice with saline phosphate buffer to precipitate the bacterial cells. The spectrophotometer was set to a wavelength of 600 nm; turbidity was prepared from the microbial suspension with an absorption of 0.72 ± 0.03 equal to 1 × 10^9^ CFU/mL bacterial content. For LAB counts before and after treatment with aflatoxin, a novel selective medium modified with rhamnose 2,3,5‐triphenyl tetrazolium chloride–LBS–vancomycin (M‐RTLV) was used as a selective medium for LRs. The LA was grown in the final clindamycin and ciprofloxacin concentrations of MRS‐clindamycin‐ciprofloxacin (MRS‐CC). MRS agar supplemented with vancomycin (20 mg/L, pH 5.6) and MRS supplemented with 4 μg/mL erythromycin were used as selective media for LP and LRE, respectively (Gandomi et al. 2016; Ganje et al. 2024; Sakai et al. 2010; Süle et al. 2014; Veselá et al. 2019).

### 2.6 Preparation of Yogurt, FDD‐LA and PDM

**FIGURE 2:**
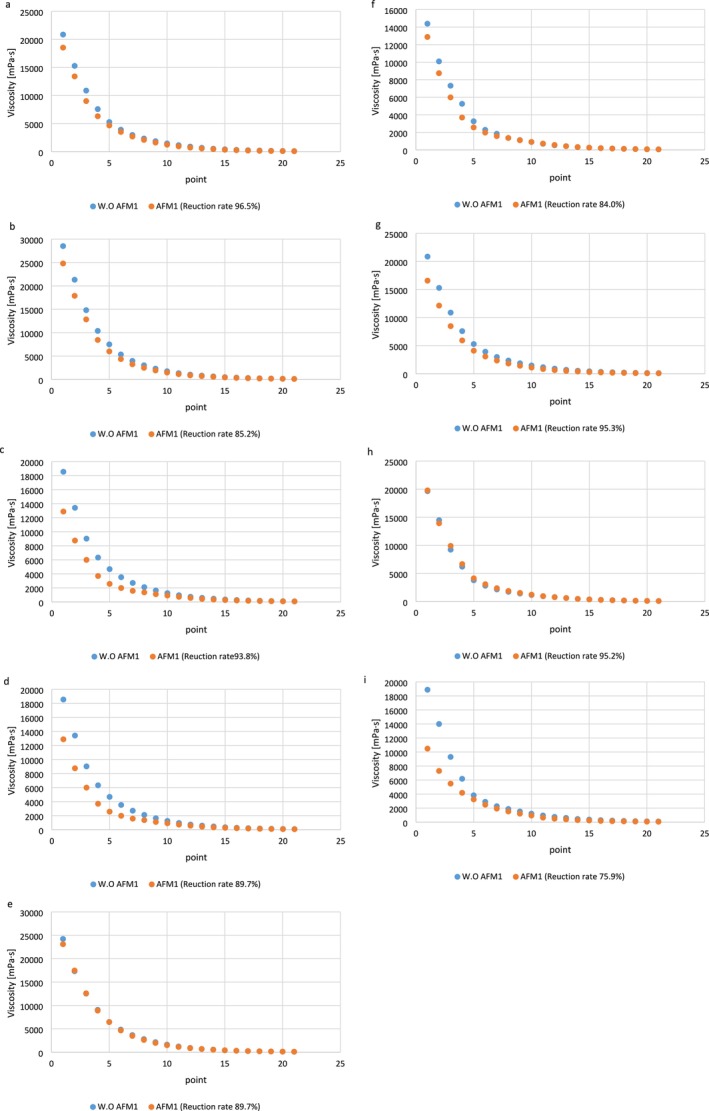
Relationship between viscosity (mPa/s) and point in PDM_LP_ (a); PDM_LRE_ (b); PDM_LR_ (c); PDM_LP‐LRE_ (d); PDM_LRE‐LR_ (e); PDM_LR‐LP_ (f); PDM_LRE‐LR‐LP_ (g); Yogurt (h); FDD‐LA (i), AFM1, Aflatoxin M1; FDD‐LA, fermented dairy drink containing *Lactobacillus acidophilus* ; LA, *Lactobacillus acidophilus* ; LP, *Lactobacillus plantarum* ; LR, *Lactobacillus rhamnosus* ; LRE, *Lactobacillus reuteri* ; PDM, probiotic drink milk; PDM_LP_, PDM contains LP; PDM_LP‐LRE_, PDM contains LP and LRE; PDM_LR_, PDM contains LR; PDM_LRE_, PDM contains LRE; PDM_LRE‐LR_, PDM contains LRE and LR; PDM_LRE‐LR‐LP_, PDM contains LRE, LR and LP; PDM_LR‐LP_, PDM contains LRE and LP; Yogurt, contains starter culture. Without aflatoxin (W.O AFM1) and with aflatoxin M1 (AFM1) in samples.

**FIGURE 3:**
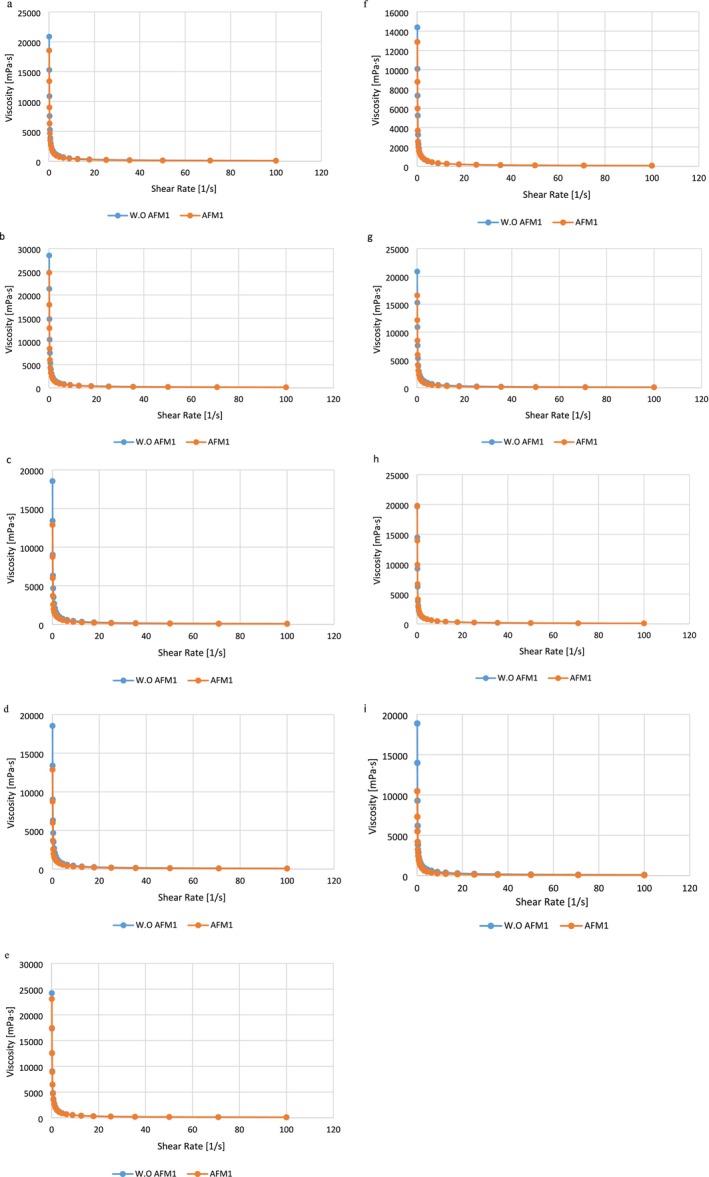
Relationship between viscosity (mPa/s) Shear rate (1/s) in PDM_LP_ (a); PDM_LRE_ (b); PDM_LR_ (c); PDM_LP‐LRE_ (d); PDM_LRE‐LR_ (e); PDM_LR‐LP_ (f); PDM_LRE‐LR‐LP_ (g); Yogurt (h); FDD‐LA (i), AFM1, Aflatoxin M1; FDD‐LA, fermented dairy drink containing *Lactobacillus acidophilus* ; LA, *Lactobacillus acidophilus* ; LP, *Lactobacillus plantarum* ; LR, *Lactobacillus rhamnosus* ; LRE, *Lactobacillus reuteri* ; PDM, probiotic drink milk; PDM_LP_, PDM contains LP; PDM_LP‐LRE_, PDM contains LP and LRE; PDM_LR_, PDM contains LR; PDM_LRE_, PDM contains LRE; PDM_LRE‐LR_, PDM contains LRE and LR; PDM_LRE‐LR‐LP_, PDM contains LRE, LR and LP; PDM_LR‐LP_, PDM contains LRE and LP; Yogurt, contains starter culture. Without aflatoxin (W.O AFM1) and with aflatoxin M1 (AFM1) in samples.

**FIGURE 4:**
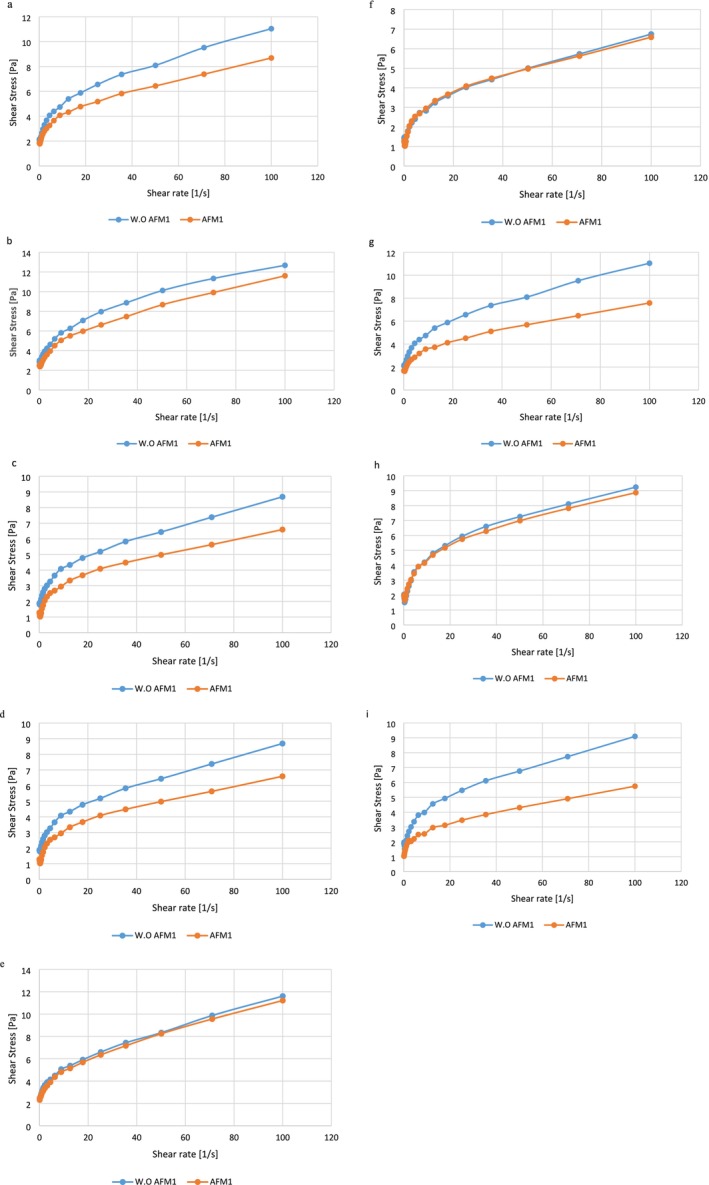
Relationship between Shear rate (1/s) and Shear stress (Pa) in PDM_LP_ (a); PDM_LRE_ (b); PDM_LR_ (c); PDM_LP‐LRE_ (d); PDM_LRE‐LR_ (e); PDM_LR‐LP_ (f); PDM_LRE‐LR‐LP_ (g); Yogurt (h); FDD‐LA (i), AFM1, Aflatoxin M1; FDD‐LA, fermented dairy drink containing *Lactobacillus acidophilus* ; LA, *Lactobacillus acidophilus* ; LP, *Lactobacillus plantarum* ; LR, *Lactobacillus rhamnosus* ; LRE, *Lactobacillus reuteri* ; PDM, probiotic drink milk; PDM_LP_, PDM contains LP; PDM_LP‐LRE_, PDM contains LP and LRE; PDM_LR_, PDM contains LR; PDM_LRE_, PDM contains LRE; PDM_LRE‐LR_, PDM contains LRE and LR; PDM_LRE‐LR‐LP_, PDM contains LRE; LR and LP; PDM_LR‐LP_, PDM contains LRE and LP; Yogurt, contains starter culture. Without aflatoxin (W.O AFM1) and with aflatoxin M1 (AFM1) in samples.

For the production of yogurt, FDD‐LA, and PDM, UHT cow skim milk (1.5% fat) was used, based on (Begunova et al. 2020; de Fátima Bimbatti et al. 2024; Yadav et al. 2019; Zhang et al. 2022). The production procedures of yogurt, FDD‐LA, and PDM are illustrated in Figures 2s–4s.

### 2.7 Probiotic Viability

LAB viability was evaluated via serial dilution and the pour plate method on selective media. All samples were analyzed by counting on selective media and incubating anaerobically at 37°C for 48 h. The results are presented as log CFU/mL (Gandomi et al. 2016; Ganje et al. 2024; Sakai et al. 2010; Süle et al. 2014; Veselá et al. 2019).

### 2.8 pH and Acidity

A pH meter (Greisinger Electronic, Germany) was used to measure pH. Titratable acidity was determined according to the methodology described in AOAC (2016).

### 2.9 Sem

The lyophilized samples were fixed onto an aluminum holder and subsequently coated with gold via a Desk Sputter Coater DSR1 (Nanostructural Coating Co., Iran) before examination under a scanning electron microscope (SEM, VEGA3, TESCAN, Czech Republic). The examination was carried out at a voltage of 0.10 kV. The distance between the microscope lens and the sample surface was maintained at 8.91–7.03 mm throughout the process.

### 2.10 Rheology

Rheological analyses were performed on samples stored at 27°C via a rheometer (MCR 302, Anton Paar, Austria). The sample in the concentric cylinder geometry was 5 mL at 23°C, and the shear rate ranged from 10 to 100 s^−1^. The device was calibrated at 25°C for 10 min. The shear rate increased linearly from 1 to 300 s^−1^ over a duration of 3 min. To describe the flow behavior of the sample mixture, four apparent viscosity models were evaluated, with the best fitting model selected based on the highest fit values. To describe the flow behavior of the sample mixture, four apparent viscosity equations were used, with the best equation selected based on the highest fitness values. The viscosity of the samples was measured at 27°C.

### 2.11 Statistical Analysis

All tests were performed in a completely random block design and repeated three times. Comparison of means was performed with Duncan's multiple range test at a significance level of 5%. Data are reported as means ± standard deviations. Additionally, the quantitative data were statistically analyzed using SPSS, version 21.

## 3 Results and Discussion

### 3.1 Levels of AFM_1_ in Milk and Fermented Milk Products

AFM1 levels in 10 selected samples from different livestock farms in Shiraz, Fars, Iran, were measured using HPLC. The results indicated that AFM1 levels were within the permissible limit (Figure 5s). To continue the relevant tests, fifty milliliters of milk were spiked with 100 μL of a 0.5 μg/mL AFM1 solution to obtain a concentration of 1 ppb of milk. Table 1 displays the AFM1 levels in the PDM, FDD‐LA, and yogurt samples (Figure 6s). The lowest AFM1 residue was found in the PDM_LP_ sample (0.035 ppb) (Table 1). All probiotics reduced aflatoxin levels in the produced samples. Zareie et al. (2024) reported that reducing the AFM1 concentrations occurs through physical interactions between aflatoxin molecules and the cell wall of probiotics. Research had shown that the presence of AFM1 did not decrease LAB count (Zareie et al. 2024) (Table 2).

**TABLE 1:** Relative proportion and viable count of bacteria used in PDM, FDD‐LA, yogurt and C samples.

Treatments	Probiotic or culture	The relative proportion of the bacteria employed	Viable count (log CFU/g)
PDM_LP_	PDM contains *Lactobacillus plantarum*	—	8.25
PDM_LRE_	PDM contains *Lactobacillus reuteri*	—	8.07
PDM_LR_	PDM contains *Lactobacillus rhamnosus*	—	8.44
PDM_LP‐LRE_	PDM contains LP and LRE	^a^ 1:1	8.13
PDM_LRE‐LR_	PDM contains LRE and LR	^a^ 1:1	8.25
PDM_LR‐LP_	PDM contains LRE and LP	^a^ 1:1	8.14
PDM_LRE‐LR‐LP_	PDM contains LRE, LR and LP	^b^ 1:1:1	8.15
Yogurt	LB and ST	^a^ 1:1	12.02
FDD‐LA	Contains *Lactobacillus acidophilus*	—	8.16

Abbreviations: FDD‐LA, fermented dairy drink containing *Lactobacillus acidophilus* ; LB, *Lactobacillus bulgaricus* ; LP, *Lactobacillus plantarum* ; LR, *Lactobacillus rhamnosus* ; LRE, *Lactobacillus reuteri* ; PDM, probiotic drink milk; ST, Streptococcus thermophiles.

^a^ The ratio of the two bacteria species was 1:1, and the total number of bacteria was approximately 8 (log CFU/g).

^b^ The ratio of the three bacterial species was 1:1:1, and the total number of bacteria was approximately 8 (log CFU/g).

**TABLE 2:** AFM1 residue in samples after fermentation.

Samples	AFM1 (ppb)
PDM_LP_	0.035
PDM_LRE_	0.148
PDM_LR_	0.062
PDM_LP‐LRE_	0.103
PDM_LRE‐LR_	0.103
PDM_LR‐LP_	0.160
PDM_LP‐LRE‐LR_	0.047
FDD‐LA	0.241
Yogurt	0.048

*Note:* AFM1 was artificially contaminated in each sample was 1 ppb and amount of aflatoxin in primary milk was zero.

Abbreviations: AFM1; Aflatoxin M1; FDD‐LA; fermented dairy drink containing *Lactobacillus acidophilus* ; LA; *Lactobacillus acidophilus* ; LP; *Lactobacillus plantarum* ; LR; *Lactobacillus rhamnosus* ; LRE; *Lactobacillus reuteri* ; PDM; probiotic drink milk; PDM_LP_; PDM contains LP; PDM_LP‐LRE_; PDM contains LP and LRE; PDM_LR_; PDM contains LR; PDM_LRE_; PDM contains LRE; PDM_LRE‐LR_; PDM contains LRE and LR; PDM_LRE‐LR‐LP_; PDM contains LRE; LR and LP; PDM_LR‐LP_; PDM contains LRE and LP; Yogurt; contains starter culture.

Moreover, the combination of probiotics reduced the amount of AFM1 in the product. Similarly, Erfanpoor et al. (2024) investigated the efficiency of LP and *Lactobacillus brevis* and their combinations in reducing AFM1 in milk. After 3 days of storage at 4°C, the AFM1 residue levels in *L. brevis* and LP were 0.052 ± 0.002 ng/mL and 0.074 ± 0.003 ng/mL, respectively, while the milk sample free of probiotic bacteria contained 0.116 ± 0.0039 ng/mL AFM1 under the same conditions. The range of reduction of AFM1 in milk by the combination of LP and *L. brevis* was 0.065 ± 0.002 ng/mL (Erfanpoor et al. 2024). This result was inconsistent with the conclusions of the current investigation. The differences in AFM1 binding capacity across different types of *lactobacilli* may help explain some of the discrepancies noted. Although variations exist in AFM1 characteristics among the different types of *lactobacilli* and the responses of the starter culture used, these inconsistencies are likely due to factors such as the population of bacteria or fermentation conditions. Consequently, the percentage of AFM1 removal has varied considerably, with rates ranging from below 10% to 100% (Zareie et al. 2024).

For the FDD‐LA sample, the rate of AFM1 reduction was 76%. The results of these studies indicated that *L. acidophilus* strains can eliminate AFB1 at high concentrations (> 50%) (Mahmood Fashandi et al. 2018). Moreover, Sokoutifar et al. (2018) reported that the maximum percentage of AFM1 removed from Doogh (a traditional Iranian fermented milk with yogurt culture alone) was 76.30% ± 0.23% at 4°C after 30 days of storage, which is consistent with the results of our study (Sokoutifar et al. 2018).

In this study, the AFM1 residue in yogurt was 0.048 ppb. One reason for the reduction in aflatoxin content may be related to the addition of *Lactobacillus bulgaricus* and *Streptococcus thermophilus* to the milk. The bonds formed between protein and AFM1 are reduced as a result of the accelerated fermentation process, coupled with an increase in proteolytic activity in yogurt (Sanaldi and Coban 2023).

Moreover, in fermented dairy products, such as yogurt, the AFM1 level was significantly reduced. A reduction of 73.6% in AFM1 was noted after 4 h of incubation at 45°C when yogurt was used. This phenomenon could be attributed to the decrease in pH that occurs with extended incubation, potentially affecting both the coagulation and denaturation of casein. Consequently, these changes could further influence the adsorption characteristics of AFM1 (Wang et al. 2023).

AFM1 reduction levels in PDM_LP_, PDM_LRE_, and PDM_LR_ were 95%, 85.2%, and 69.0%, respectively. Further, PDM_LP‐LRE_, PDM_LRE‐LR_, and PDM_LR‐LP_ achieved reductions of 84%, 84%, and 89.7%, respectively. Research examining the impact of probiotics on the reduction in aflatoxins revealed that, after a 24 h incubation period, LRs and LPs reduced AFM1 to 78.70% and 50.50%, respectively. The findings of this study do not align with those of previous studies (Luqman et al. 2024). The differences noted between the findings of this study and those from previous studies may be attributed to variations in fermentation time, probiotic concentration, aflatoxin concentration, and the temperature of fermentation. The fermentation time in Luqman et al.'s (2024) study was 24 h, which is greater than in our research (4 h). As a result, the decrease in AFM1 observed in their research was greater than ours. Increased AFM1 reduction percentages with increasing incubation temperatures were also observed. This is consistent with the role of fermentation and LAB growth as key players in AFM1 removal. Similar results were found in Shigute and Washe (2018) research. Moreover, the variation in results among the different probiotic strains indicates that various binding sites are associated with different strains involved in aflatoxin removal (Luqman et al. 2024). Based on the literature review, the binding of aflatoxin using probiotic microorganisms is fast and possibly reversible. However, some factors such as toxin and bacteria concentration directly influence the aflatoxin binding rate (Emadi et al. 2022). Fermentation temperature influences aflatoxins removal. Fermentation can reduce the amount and toxicology of free aflatoxins by structural decomposition of aflatoxins due to low pH and the biological activity of starter microorganisms. This activity enhances the binding capacity of aflatoxins to milk proteins due to structural changes caused by pH reduction and acid formation, as well as binding of aflatoxins to the bacteria cells (Arab et al. 2012).

Optimal pH (e.g., acidic conditions in fermented milk) enhances bacterial activity and AFM1 binding. Acidity formed during the fermentation enhances the ability of microorganisms to bind the AFM1. These results are also in agreement with the results obtained by (Elsanhoty et al. 2014).

Additionally, changes in milk protein structure, such as the formation of yogurt coagulum from casein, result from the decrease in pH during fermentation. This structural change affects the AFM1 association with this protein, leading to adsorption or occlusion of the toxin in the precipitate. LAB have the ability to bind with AFM1, thus resulting in the reduction of free AFM1 content during yogurt processing (Tahoun et al. 2017). *Lactobacilli* concentration is one of the factors affecting the AFM1 reduction rate (Ismail et al. 2017). Additionally, the methods used for AFM1 analysis, like HPLC, ELISA, LC‐MS/MS, and LC‐IDMS do not have the same sensitivity (Kos et al. 2016; Tölgyesi et al. 2022).

Factors such as AFM1 concentration, probiotic density, temperature, duration of exposure to probiotics, microbial strains, and exposure time are major factors affecting aflatoxin reduction. Moreover, microbial cell wall polysaccharides and peptidoglycans can influence the AFM1 reduction (Rezasoltani et al. 2022). Probiotic growth is another parameter; microbial growth also depends on environmental factors such as pH, temperature, and accumulation of metabolic end‐products. Several investigators have demonstrated the effects of these parameters on the growth of LAB based on experiments (Charalampopoulos et al. 2002).

In this study, we aimed to control certain environmental factors. Specific growth rate and microbial strains were two important factors affecting AFM1 reduction. Besides genetic diversity, mechanisms of probiotic action, dairy processing methods, and synergistic interactions can also affect aflatoxin reduction. A previous study revealed that variations in the rate of AFB1 degradation depend on the probiotic type, enzyme state, microbial action, and more specifically, acid coagulation led to the degradation of AFM1 in milk (Elsanhoty et al. 2014).

### 3.2 TVNP

The TVNPs and starter cultures of samples with and without AFM1 after production are presented in Table 3. The TVNP concentration was 8 log CFU/g at the beginning of production. In all samples, the TVNP increased during production. However, the TVNPs of the samples treated with AFM1 were slightly lower than those of the other samples, although no statistically significant differences were observed among the samples. The findings of other studies also support this point; for instance, no significant changes in TVNP were detected with AFM1 treatment (Adácsi et al. 2023). One reason for the difference in the survival of *Lactococcus lactis* compared with our study may be the variation in the amount of aflatoxin used in this research.

**TABLE 3:** Total viable number of probiotic bacteria in PDM, C, FDD‐LA, and yogurt after processing period.

Samples without AFM1	Viable number of probiotic (log CFU/g)	Samples with AFM1	Viable number of probiotic (log CFU/g)
PDM_LP_	12.51 ± 0.62^A^	PDM_LP_	12.48 ± 0.58^A^
PDM_LRE_	12.2 ± 0.50^A^	PDM_LRE_	12.33 ± 0.55^A^
PDM_LR_	12.38 ± 0.55^A^	PDM_LR_	12.40 ± 0.56^A^
PDM_LP‐LRE_	12.36 ± 0.44^A^	PDM_LP‐LRE_	11.97 ± 0.57^A^
PDM_LRE‐LR_	12.58 ± 0.47^A^	PDM_LRE‐LR_	12.63 ± 0.46^A^
PDM_LR‐LP_	12.81 ± 0.50^A^	PDM_LR‐LP_	12.34 ± 0.49^A^
PDM_LP‐LRE‐LR_	12.65 ± 0.48^A^	PDM_LP‐LRE‐LR_	12.91 ± 0.67^A^
FDD‐LA	13.12 ± 0.52^A^	FDD‐LA	12.68 ± 0.58^A^
Yogurt	12.14 ± 0.13^A^	Yogurt	12.05 ± 0.13^A^

*Note:* Data (mean ± standard deviation) are from three replications. Uppercase letters (A) show significant different (*p* ≤ 0.05) among samples in each row. The initial viable number of bacteria was approximately 8 log CFU/mL.

Abbreviations: AFM1; Aflatoxin M1; FDD‐LA; fermented dairy drink containing *Lactobacillus acidophilus* ; LA; *Lactobacillus acidophilus* ; LP; *Lactobacillus plantarum* ; LR; *Lactobacillus rhamnosus* ; LRE; *Lactobacillus reuteri* ; PDM; probiotic drink milk; PDM_LP_; PDM contains LP; PDM_LP‐LRE_; PDM contains LP and LRE; PDM_LR_; PDM contains LR; PDM_LRE_; PDM contains LRE; PDM_LRE‐LR_; PDM contains LRE and LR; PDM_LRE‐LR‐LP_; PDM contains LRE; LR and LP; PDM_LR‐LP_; PDM contains LRE and LP; Yogurt; contains starter culture.

The microbial removal of AFM1 involves cell components such as proteins, polysaccharides, and peptidoglycan, particularly the teichoic acid of the cell wall, which can bind to AFM1. The binding of aflatoxin to the cell wall or its components causes degradation of the cell wall components. The lack of specific components of the cell wall may result in a decrease in the adsorption capacity of the cells, suggesting that adsorption or adhesion depends on the proper structure of the cell wall. Therefore, a decrease in the bacterial population can be observed (Panwar et al. 2019; Zhang et al. 2019).

### 3.3 Sem

Figure 1a–t shows the differences in microstructure caused by the various treatments applied to milk and fermented milk. Significant variations were found in the microstructure properties between the AFM1 samples and control. The analysis indicated that the product matrix without AFM1 had a denser structure. Additionally, the addition of LABs could significantly affect the development or modification of the microstructure, likely due to its ability to increase body and texture. For the samples that underwent AFM1 treatment, an increase in the pore size in the network was noted. As a result, a less compacted microstructure may be the result of demineralization (loss of calcium and phosphate in the casein micelle) that occurs due to the decrease in pH (Gonçalves et al. 2021). When aflatoxin is not properly bound, its levels decrease because of the instability of casein micelles (El‐kest et al. 2015) Thus, acidic condition can influence the AFM1's interaction with casein (Mohammadi 2011). Consistent with our findings, Gonçalves et al. (2021) reported similar results in Minas Frescal cheese.

The microstructure of the fermented product is a spatial arrangement of casein micelles that join in groups and chains to form a viscoelastic protein network through which moisture, fat globules, and bacteria are dispersed. There was an increase in the network pores of the fermented product containing AFM1. Moreover, the formulation of a new protein structure may lead to increased AFM1 adsorption (Gonçalves et al. 2021).

### 3.4 pH

The pH decreased in all samples after processing (Table 4). The pH of the yogurt coagulum facilitates the adsorption of AFM1 by denaturing and coagulating casein protein. Shigute and Washe (2018) noted a significant decrease in the AFM1 concentration in fresh milk samples during fermentation of ergo. This was attributed to the growth of naturally occurring LABs and a gradual decrease in pH (Shigute and Washe 2018).

**TABLE 4:** pH in PDM, FDD‐LA, Yogurt samples with and without AFM1 after fermentation.

Samples without AFM1	pH	Samples with AFM1	pH
PDM_LP_	3.75 + 0.03A	PDM_LP_	3.73 + 0.01A
PDM_LRE_	3.75 + 0.01A	PDM_LRE_	3.72 + 0.02A
PDM_LR_	4.57 + 0.02A	PDM_LR_	4.54 + 0.02A
PDM_LP‐LRE_	3.76 + 0.01A	PDM_LP‐LRE_	3.74 + 0.03A
PDM_LRE‐LR_	3.70 + 0.06A	PDM_LRE‐LR_	3.69 + 0.02A
PDM_LR‐LP_	3.83 + 0.01A	PDM_LR‐LP_	3.81 + 0.01A
PDM_LP‐LRE‐LR_	3.86 + 0.02A	PDM_LP‐LRE‐LR_	3.82 + 0.02A
FDD‐LA	3.86 + 0.01A	FDD‐LA	3.87 + 0.03A
Yogurt	4.62 + 0.01A	Yogurt	4.61 + 0.01A

*Note:* The pH of all samples before application of bacteria was 6.8. Uppercase letters (A) show significant different (*p* ≤ 0.05) among samples in each row.

Abbreviations: AFM1; Aflatoxin M1; FDD‐LA; fermented dairy drink containing *Lactobacillus acidophilus* ; LA; *Lactobacillus acidophilus* ; LP; *Lactobacillus plantarum* ; LR; *Lactobacillus rhamnosus* ; LRE; *Lactobacillus reuteri* ; PDM; probiotic drink milk; PDM; probiotic drink milk; PDM_LP_; PDM contains LP; PDM_LP‐LRE_; PDM contains LP and LRE; PDM_LR_; PDM contains LR; PDM_LRE_; PDM contains LRE; PDM_LRE‐LR_; PDM contains LRE and LR; PDM_LRE‐LR‐LP_; PDM contains LRE; LR and LP; PDM_LR‐LP_; PDM contains LRE and LP; Yogurt; contains starter culture.

The pH of the AFM1‐treated samples did not significantly differ from that of the control samples. This may be related to the lack of significant change in the TVNP between the mentioned samples (Table 3). It was found that a decrease of pH during storage can lead to a further reduction in AFM1 levels in milk, as the development of organic acids and a reduction in pH can alter the structure of caseins and protein components. These changes can lead to the formation of a network like yogurt coagulum, which holds the aflatoxin inside the precipitate (Zareie et al. 2024). Furthermore, pH reduction may be due to the ability of strains to bind AFM1 in their cell wall. The pH affects their growth, metabolic activity, and enzyme production of probiotic bacteria. Numerous studies have reported that low pH, depletion of nutrients, and microbial competition do not explain the reason for AFM1 inhibition. Some researchers have suggested that the inhibition of AFM1 is due to lactic acid and/or LAB metabolites, which are heat‐stable low molecular weight compounds (Elsanhoty et al. 2014).

### 3.5 Rheology

The decrease in viscosity observed in all samples was accompanied by a decrease in the shear rate, as shown in Figure 3a–i, ultimately resulting in stable conditions. This pattern is typical of non‐Newtonian behavior (shear thinning). These findings align with those reported by D'Alessandro et al. (2023). All samples contained a pseudoplastic fluid, which led to a relatively high apparent viscosity due to its low shear rate (D'Alessandro et al. 2023). As the shear rate gradually increased, a slow decline in the slope of the curve was observed, indicating a diminishing trend in the viscosity of the liquid. This phenomenon may be related to a decrease in particle size that occurs with increasing shear rate (Torres et al. 2018).

The apparent viscosity of the samples without AFM1 was greater than that of the control samples. Moreover, the samples with added AFM1 showed a significant decrease in apparent viscosity in all the samples tested (Figure 2a–i). This might be due to the influence of the interaction of AFM1 with casein and the resulting changes in its structure, especially in the secondary structure (Harshitha et al. 2023). The increase in shear stress matched the increase in the shear rate (Figure 4a–i). As expected, all samples exhibited Bingham plastic behavior (Hashim et al. 2021). The differences noted between the findings of this study and those from previous studies may be attributed to variations in storage duration.

The viscosity of AFM1 treated samples was lower than that of the control samples. Reduced viscosity helps LABs to reduce AFM1. The results indicated that removing aflatoxin that is bonded or attached strongly to the inner texture of food is challenging (Jalili 2016). Reducing viscosity helps LABs lower AFM1 levels. We believe that when viscosity is lower, probiotics and AFM1 can interact more efficiently. This creates a larger surface area for probiotics to bind with toxins and enhances enzyme diffusion and activity, which ultimately supports the detoxification of AFM1.

## 4 Conclusion

The results of these studies revealed that LABs have the ability to break down AFM1, which could lead to a reduction in toxin levels to safe levels in PDM, FDD‐LA, and yogurt. This highlights the necessity of employing such mechanisms for removing AFM1 from contaminated food and feed, ultimately improving safety for consumers. The combination of probiotic bacteria could promote the removal of aflatoxin M1 from milk. Moreover, the studies presented here indicated that the AFM1‐supplemented samples did not have good textural properties. The incorporation of probiotics into dairy products has several benefits, including increasing their strong flavor and improving their nutritional value, while also helping to break down AFM1, which may be present in dairy products. Using probiotic bacteria in dairy production can significantly reduce AFM1 levels, making these products safer for consumers, especially in areas where aflatoxin risks are elevated. This approach not only encourages sustainable practices by minimizing chemical use, but also helps maintain the natural microbiota. By incorporating specific probiotics into standard dairy processes, healthier, probiotic‐rich products could be developed. Our research might lead to new regulations and guidelines for managing AFM1 effectively.

## Author Contributions

**Fatemeh Moradkhani:** investigation (lead), methodology (lead). **Seyed Saeed Sekhavatizadeh:** writing – original draft (lead), writing – review and editing (lead). **Mohammad Hosein Marhamatizadeh:** project administration (equal). **Maryam Ghasemi:** writing – review and editing (equal).

## Ethics Statement

This study does not involve any human or animal testing.

## Conflicts of Interest

The authors declare no conflicts of interest.

## Supporting information


Data S1.

## Data Availability

Data will be made available upon reasonable request.
