# Supplementary material for: Biodetoxification of Aflatoxin M1 in Artificially Contaminated Fermented Milk, Fermented Dairy Drink and Yogurt Using Lactobacillus acidophilus , Lactobacillus plantarum , Lactobacillus reuteri , and Lactobacillus rhamnosus and Its Effects on Physicochemical Properties
Source: Food Sci Nutr. 2025 Apr 24;13(4):e70175. doi: 10.1002/fsn3.70175 (PMC12021996; doi:10.1002/fsn3.70175)
Supplement: Supplementary file 1 — Data S1. [file FSN3-13-e70175-s001.docx]

Supplementry material

Figure 1s. The calibration curve of Aflatoxin M1

UHT cow skim milk

(1.5% fat)

1 ppb Aflatoxin M1

Without Aflatoxin

Aflatoxin Aflatoxin Aflatoxin

Aflatoxin Aflatoxin Aflatoxin

Heated to 90 °C for 15 minutes

Heated to 90 °C for 15 minutes

Aflatoxin Aflatoxin Aflatoxin

Aflatoxin Aflatoxin Aflatoxin

Cooled to 45 °C

Cooled to 45 °C

Aflatoxin Aflatoxin Aflatoxin

Aflatoxin Aflatoxin Aflatoxin

Inoculated with the CH1 starter culture 50 U

Inoculated with the CH1 starter culture 50 U

Aflatoxin Aflatoxin Aflatoxin

Aflatoxin Aflatoxin Aflatoxin

Incubated at 42 °C

Incubated at 42 °C

Aflatoxin Aflatoxin Aflatoxin

Aflatoxin Aflatoxin Aflatoxin

pH was reduced to 4.6.

pH was reduced to 4.6.

Aflatoxin Aflatoxin Aflatoxin

Aflatoxin Aflatoxin Aflatoxin

Store at 4⁰C for 24 h

Store at 4⁰C for 24 h

Figure 2s. Yogurt production diagram

Aflatoxin Aflatoxin Aflatoxin

Aflatoxin Aflatoxin Aflatoxin

Aflatoxin Aflatoxin Aflatoxin

Aflatoxin Aflatoxin Aflatoxin

Aflatoxin Aflatoxin Aflatoxin

Aflatoxin Aflatoxin Aflatoxin

UHT cow skim milk

(1.5% fat)

1 ppb Aflatoxin M1

Without Aflatoxin

Heated to 90 °C for 15 minutes

Heated to 90 °C for 15 minutes

Aflatoxin Aflatoxin Aflatoxin

Cooled to 37 °C

Cooled to 37 °C

Inoculated with LR, LP and LRE alone and combination

Inoculated with LR, LP and LRE alone and combination form

Aflatoxin Aflatoxin Aflatoxin

Aflatoxin Aflatoxin Aflatoxin

Incubated at 42 °C

Incubated at 42 °C

Aflatoxin Aflatoxin Aflatoxin

Aflatoxin Aflatoxin Aflatoxin

pH was reduced to 4.6.

pH was reduced to 4.6.

Aflatoxin Aflatoxin Aflatoxin

Store at 4⁰C for 24 h

Store at 4⁰C for 24 h

Figure 3s. Probiotic drink milk with LP=Lactobacillus plantarum, LRE=Lactobacillus reuteri, LR= Lactobacillus rhamnosus diagram

Aflatoxin Aflatoxin Aflatoxin

UHT cow skim milk

(1.5% fat)

1 ppb Aflatoxin M1

Without Aflatoxin

Aflatoxin Aflatoxin Aflatoxin

Milk preheated 37°C

Milk preheated 37°C

Aflatoxin Aflatoxin Aflatoxin

Aflatoxin Aflatoxin Aflatoxin

Inoculated with LA

Inoculated with LA

Incubated at 42 °C

Incubated at 42 °C

pH was reduced to 4.6

pH was reduced to 4.6

Store at 5⁰C for 24 h

Aflatoxin Aflatoxin Aflatoxin

Aflatoxin Aflatoxin Aflatoxin

Aflatoxin Aflatoxin Aflatoxin

Aflatoxin Aflatoxin Aflatoxin

Aflatoxin Aflatoxin Aflatoxin

Aflatoxin Aflatoxin Aflatoxin

Store at 5⁰C for 24 h

Aflatoxin Aflatoxin Aflatoxin

Aflatoxin Aflatoxin Aflatoxin

concentrated juice was mixed

concentrated juice was mixed

Figure 4s. Fermented dairy drink containing LA=Lactobacillus acidophilus


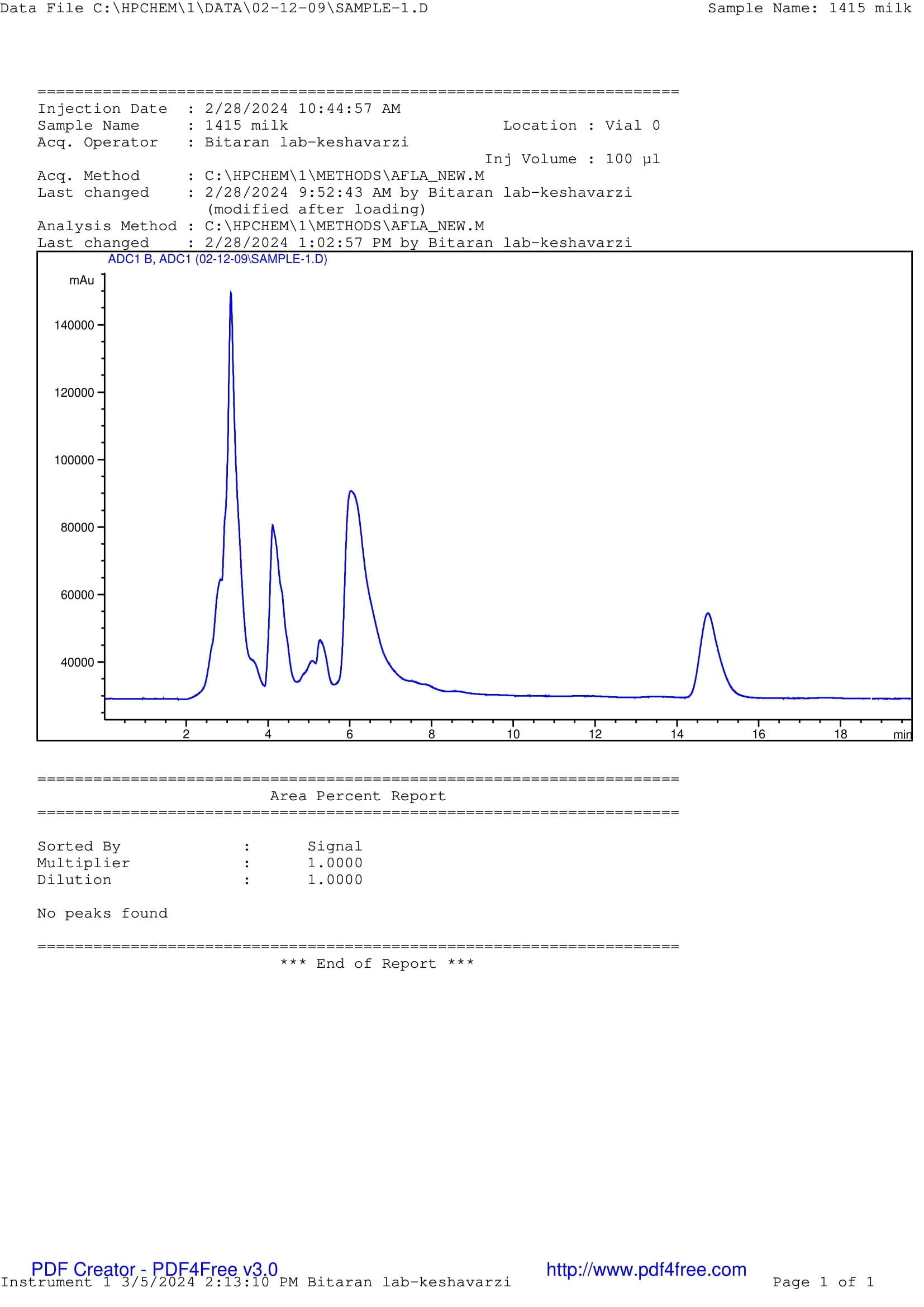


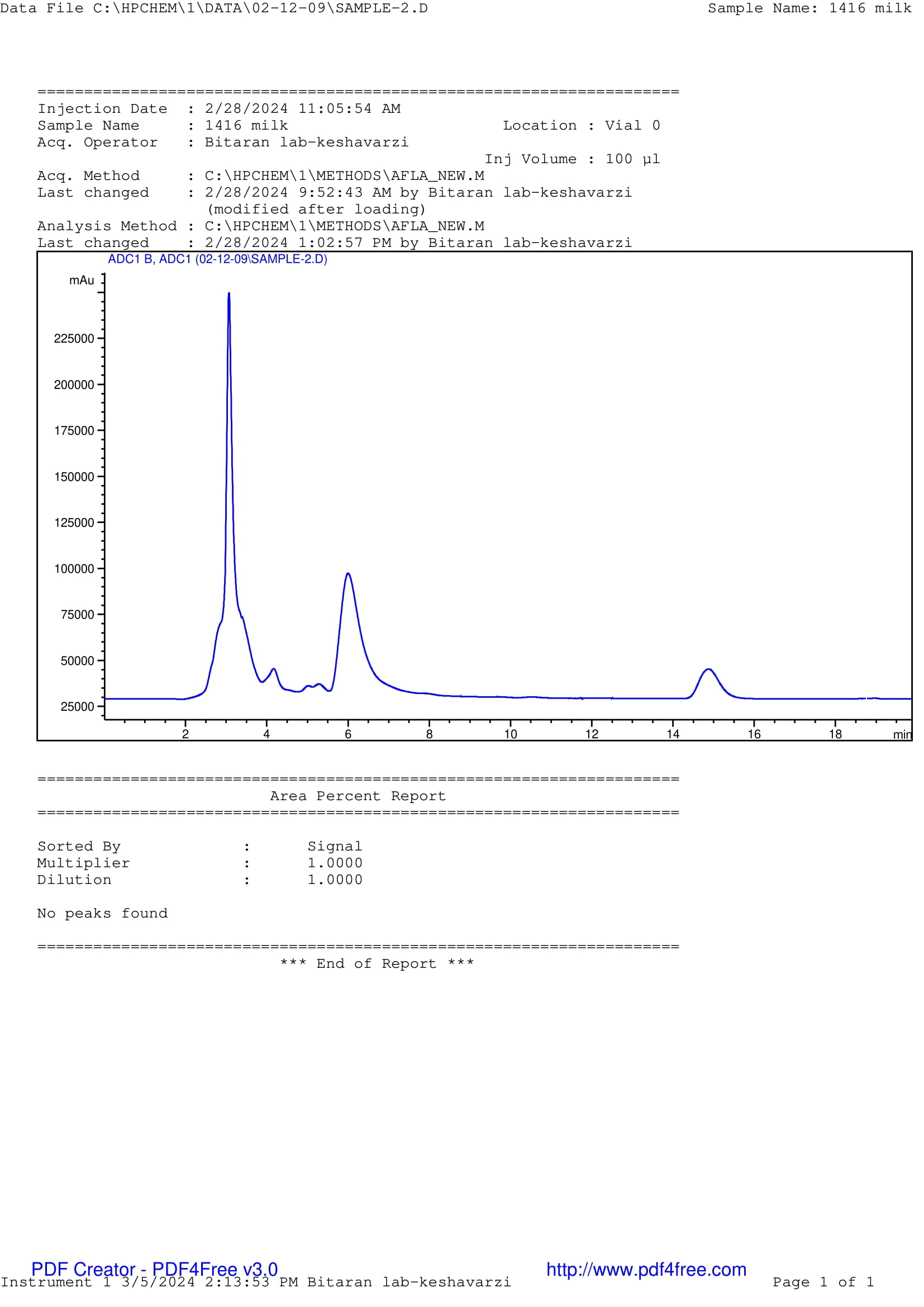


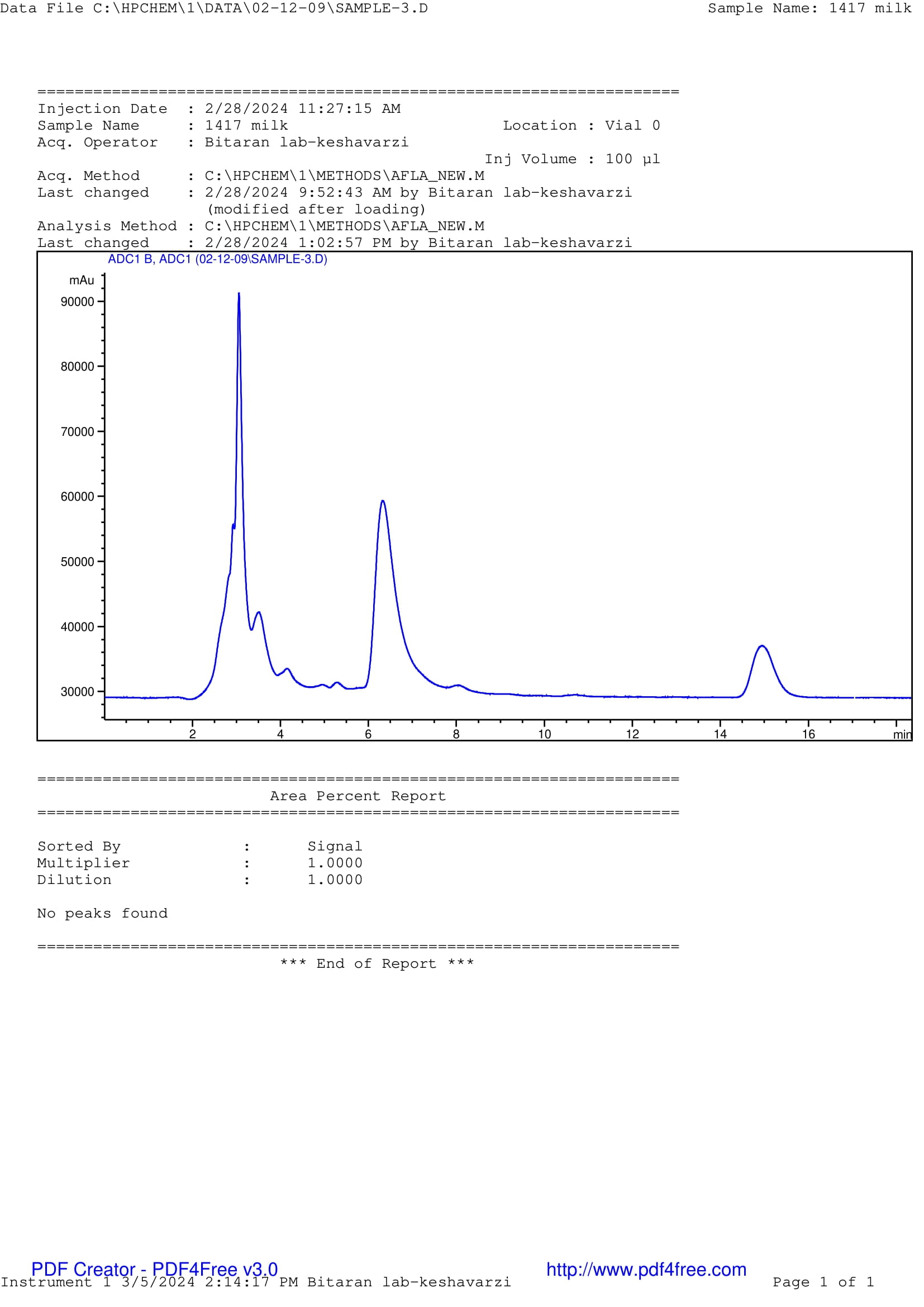


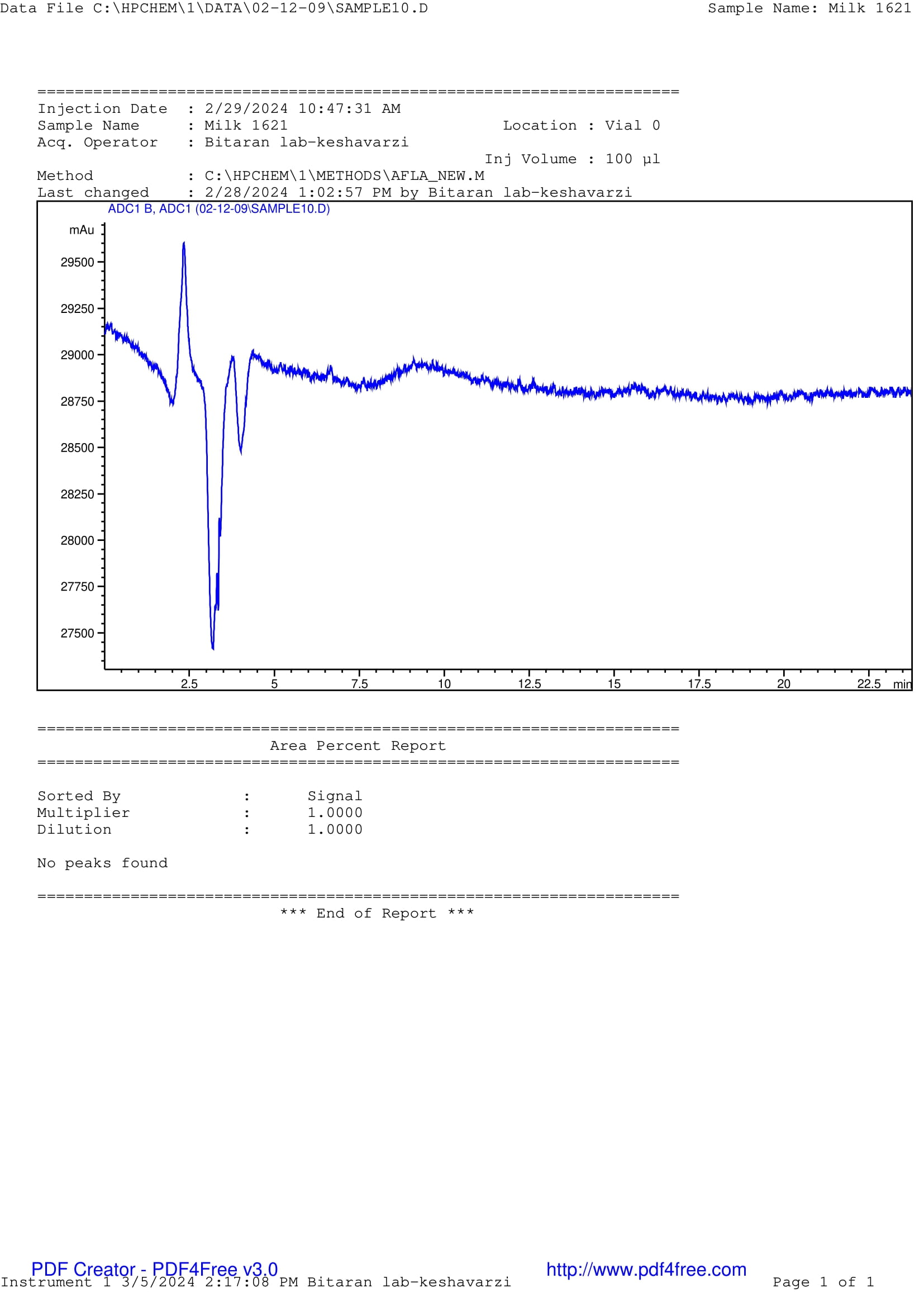


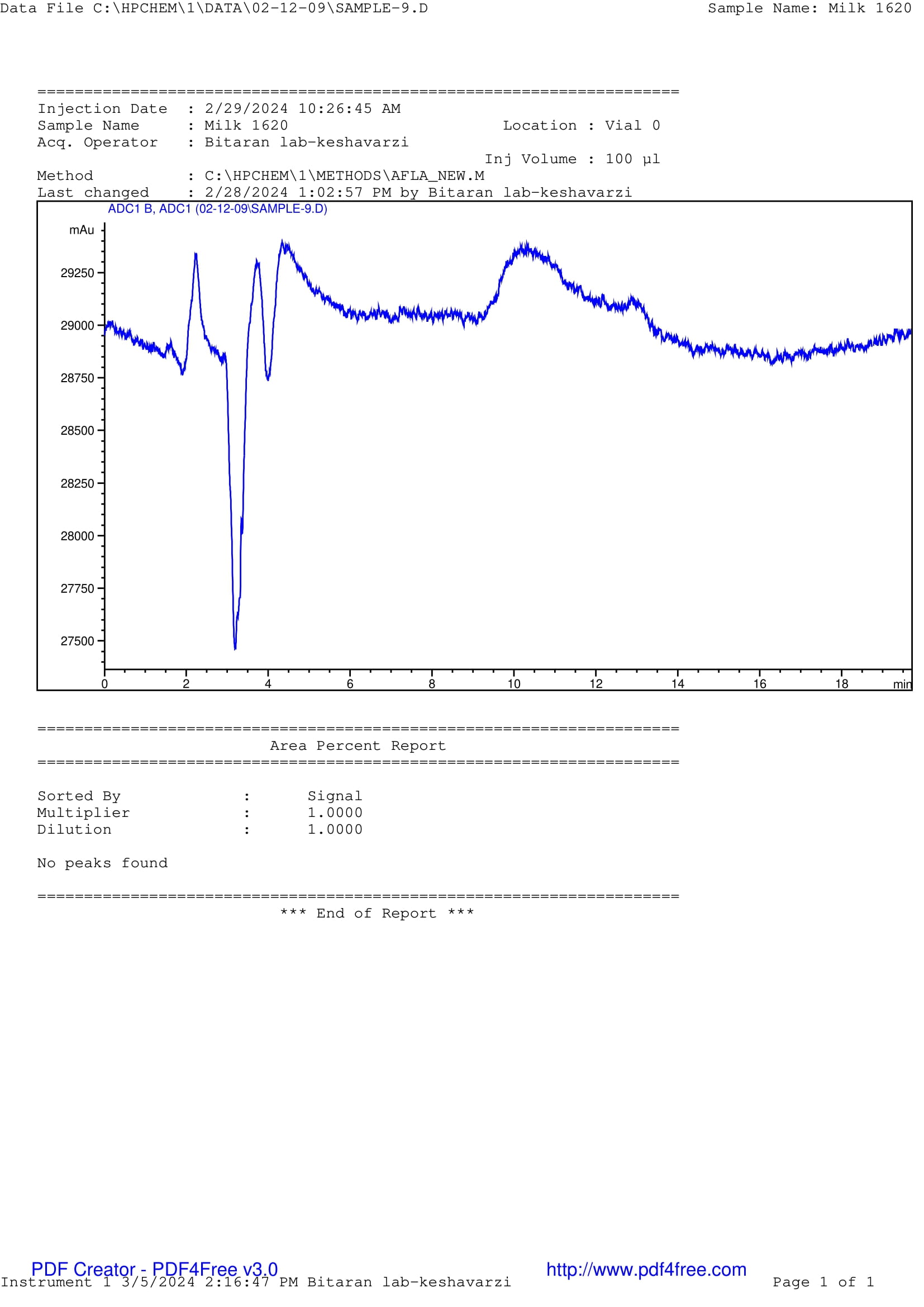

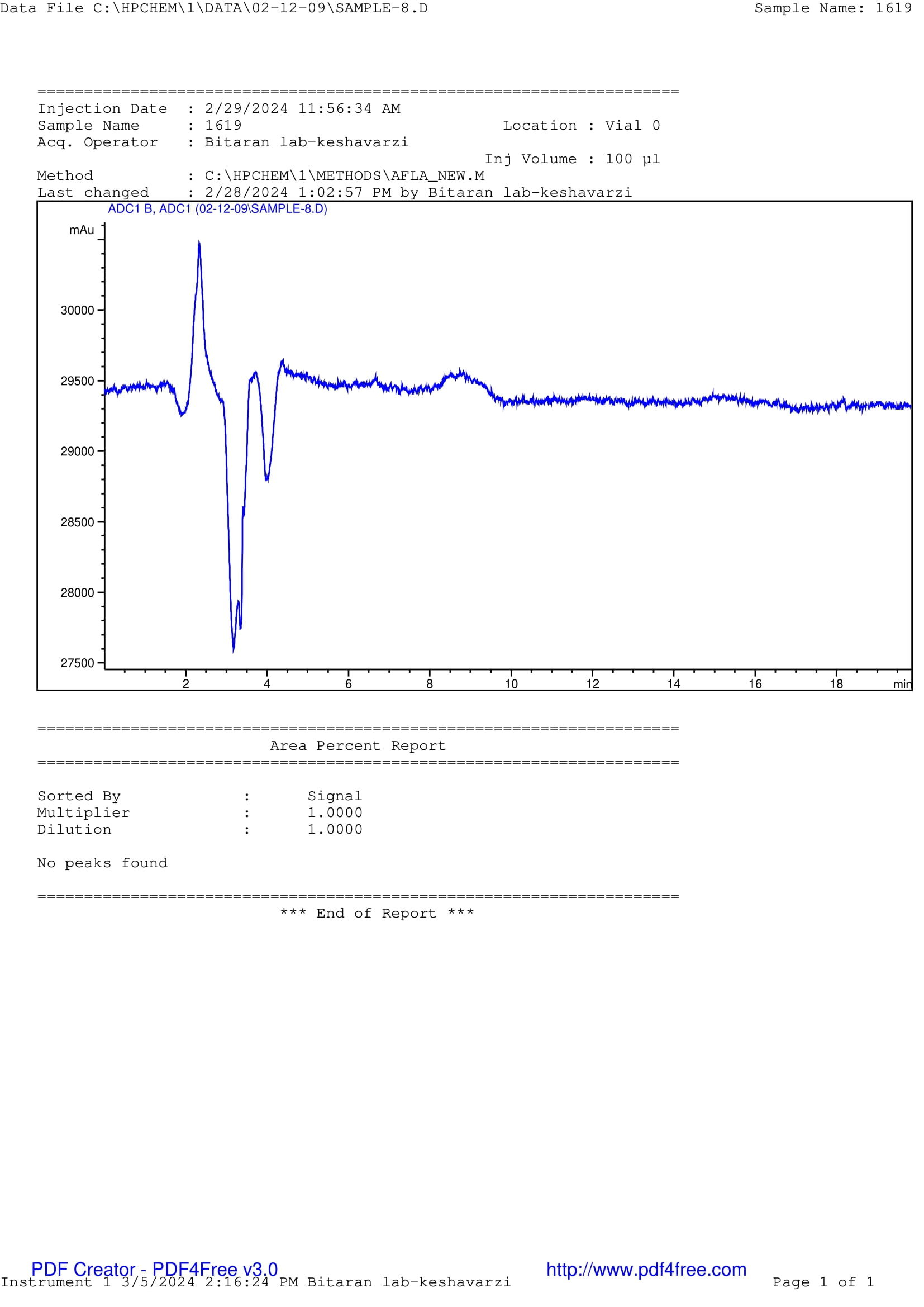


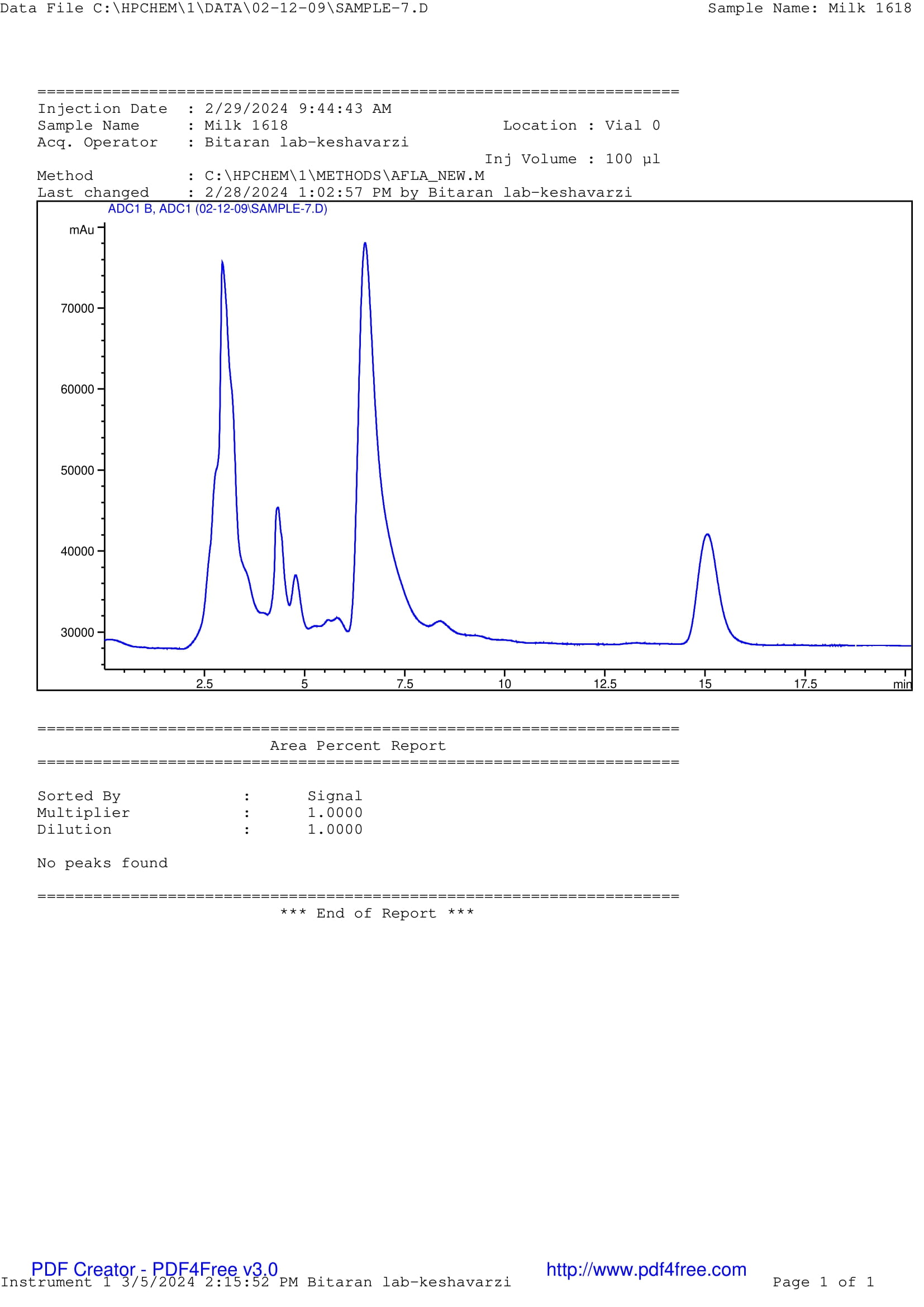

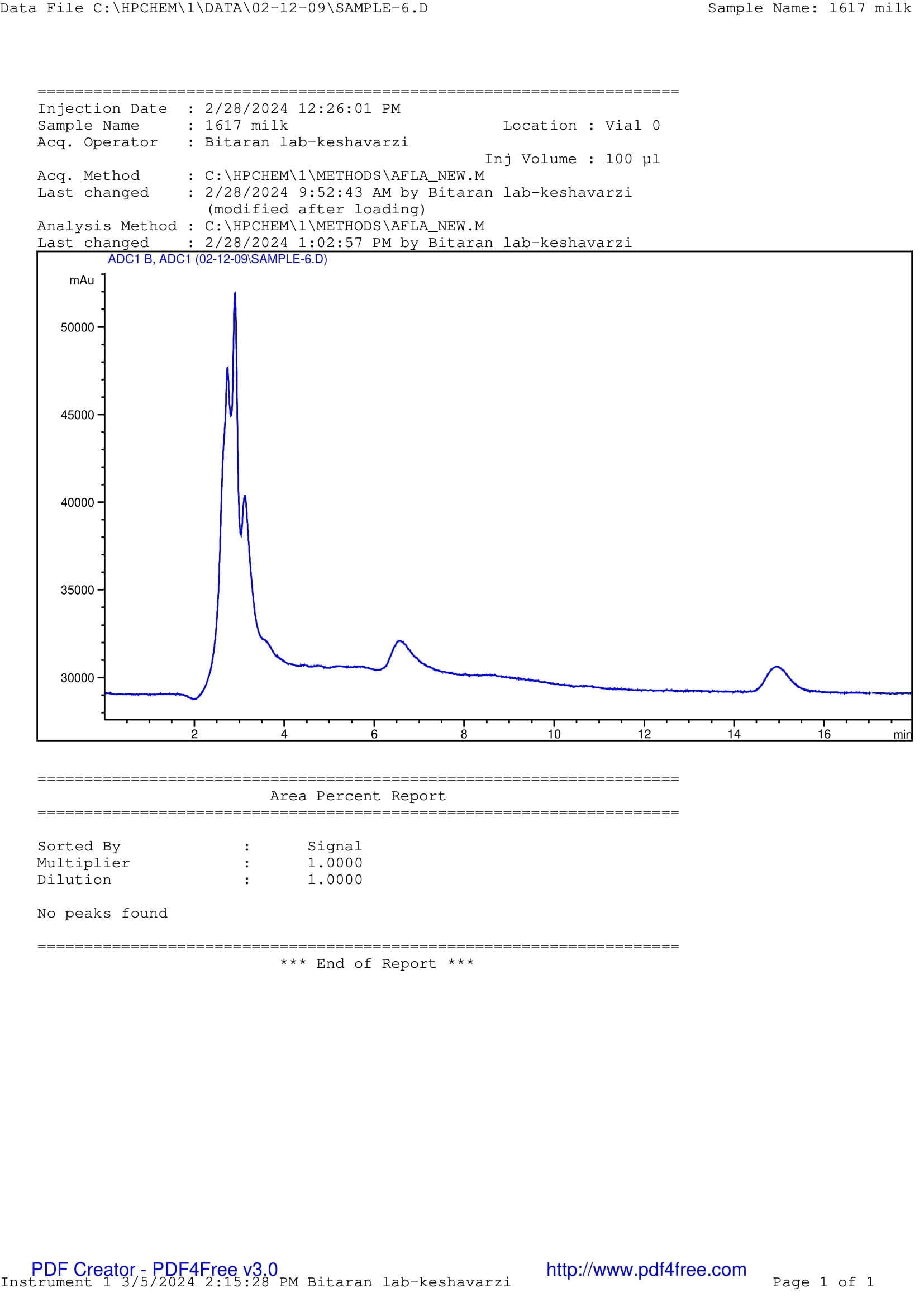


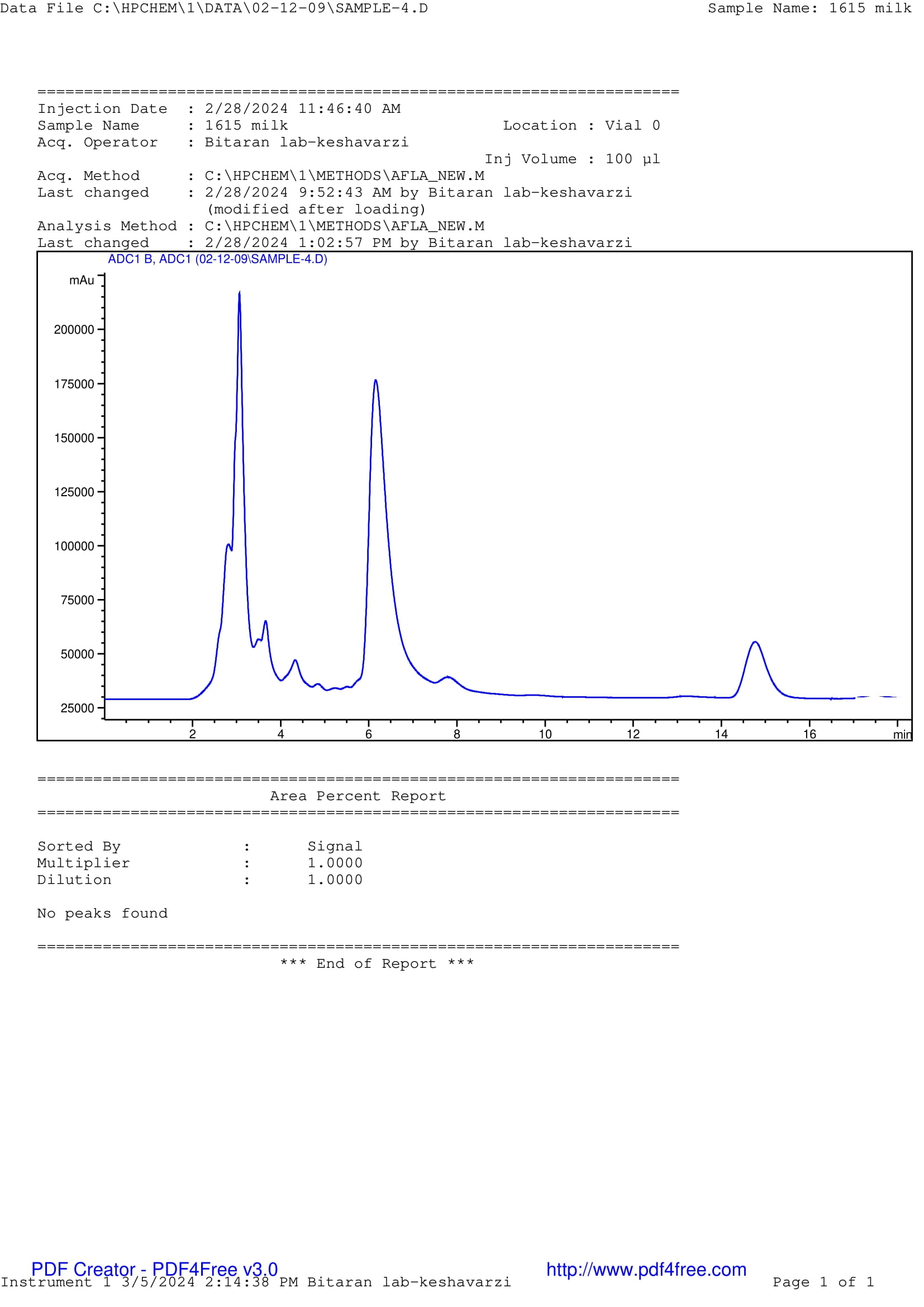

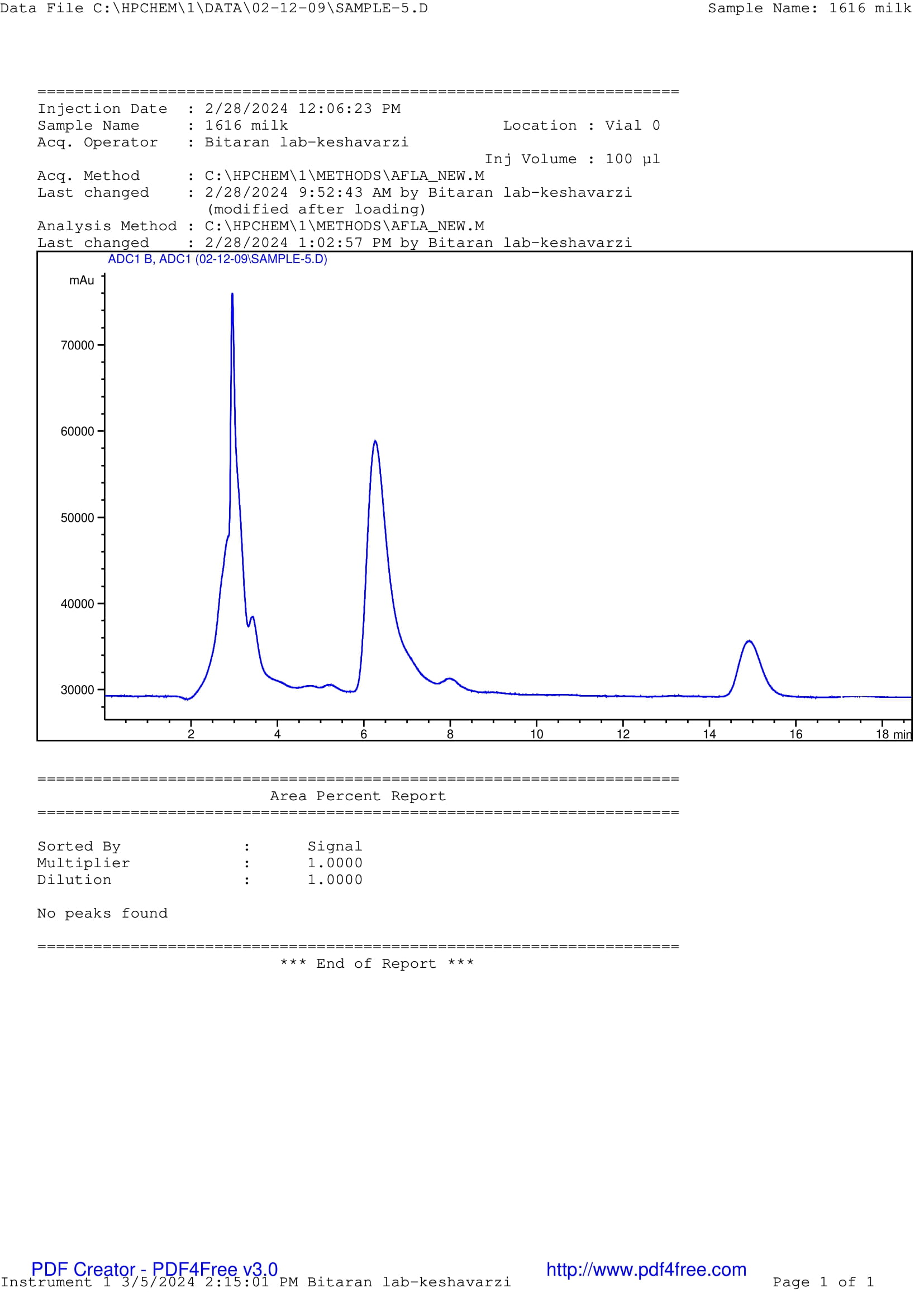


Figure 5s. HPLC choromatogram of 10 milk samples collected in Shiraz, Fars, Iran.


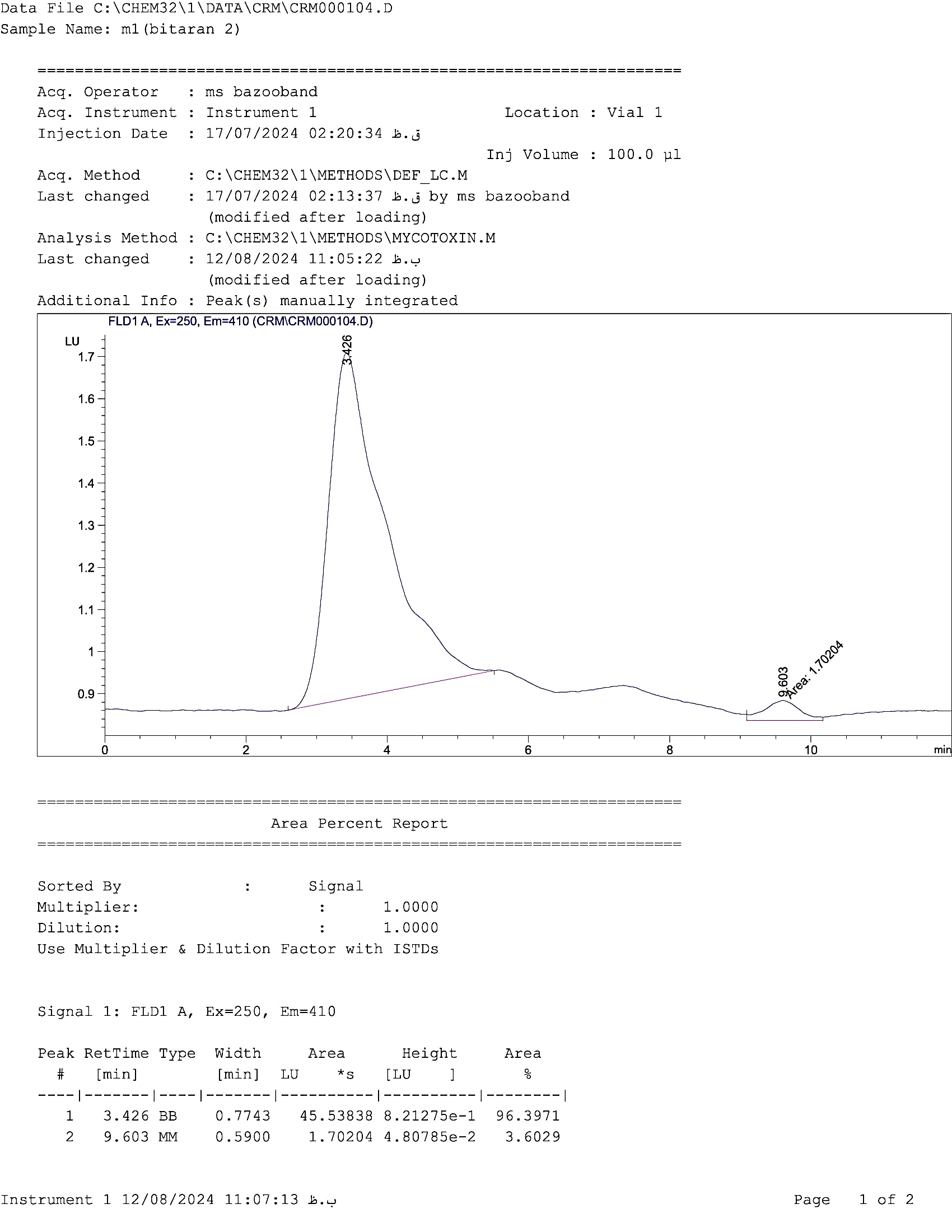


a

b


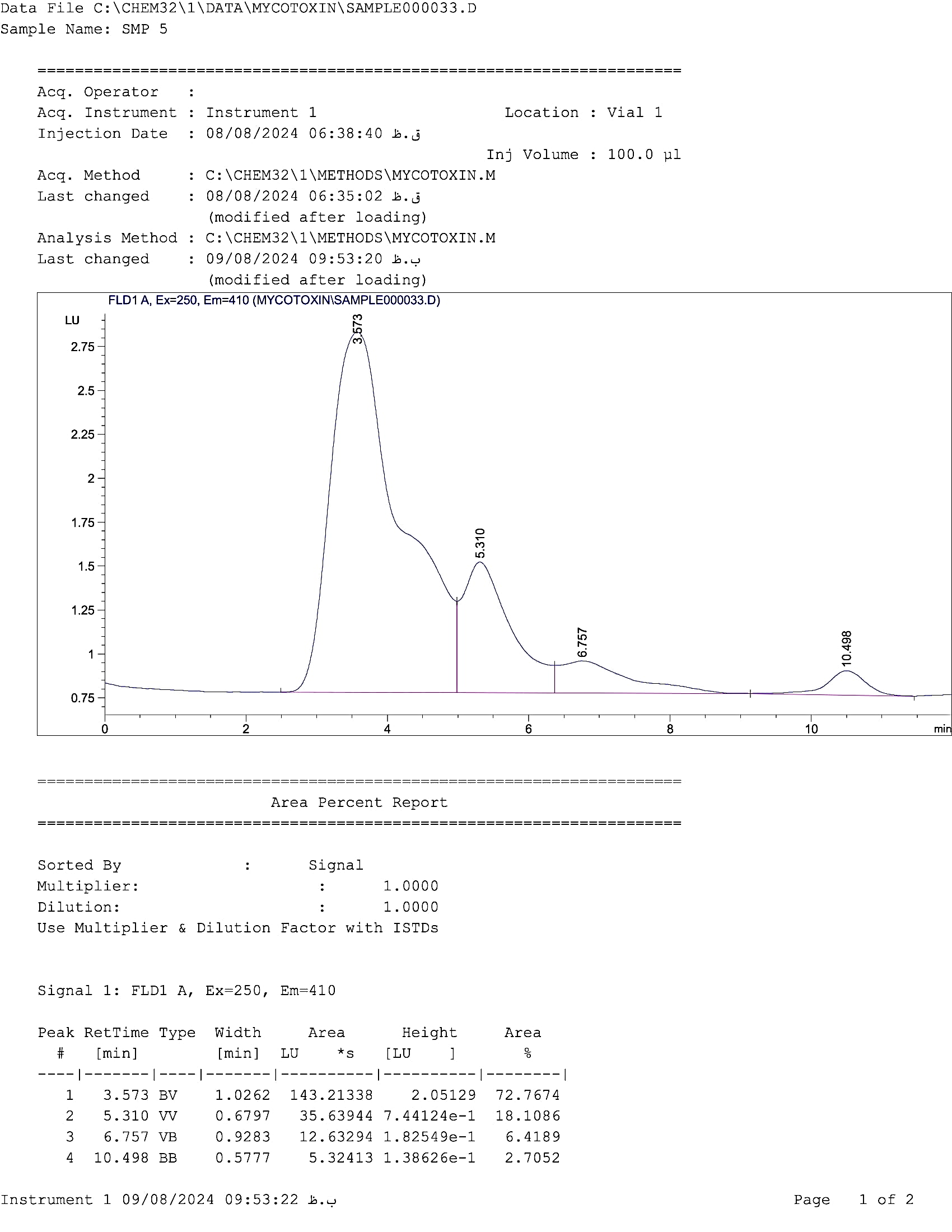


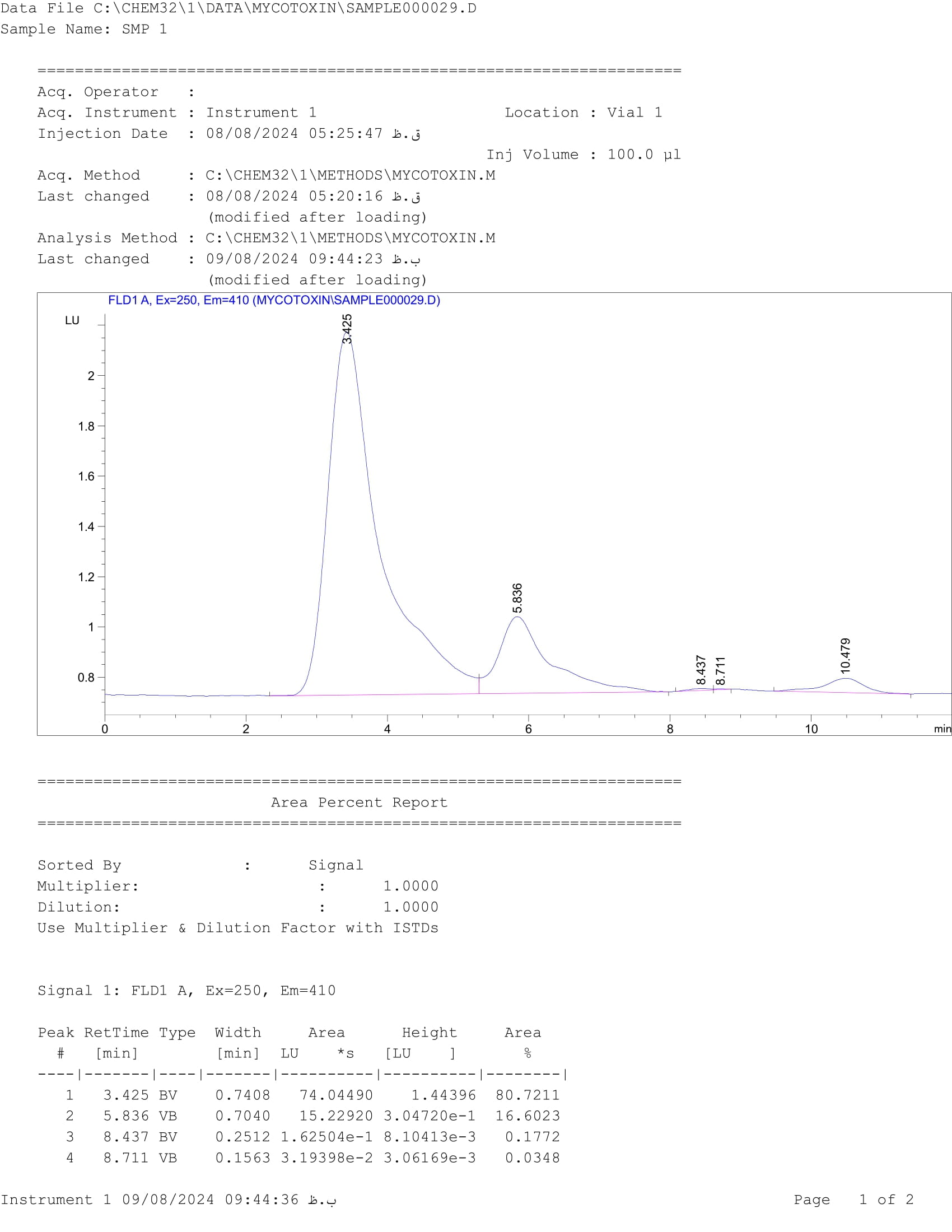


c

d


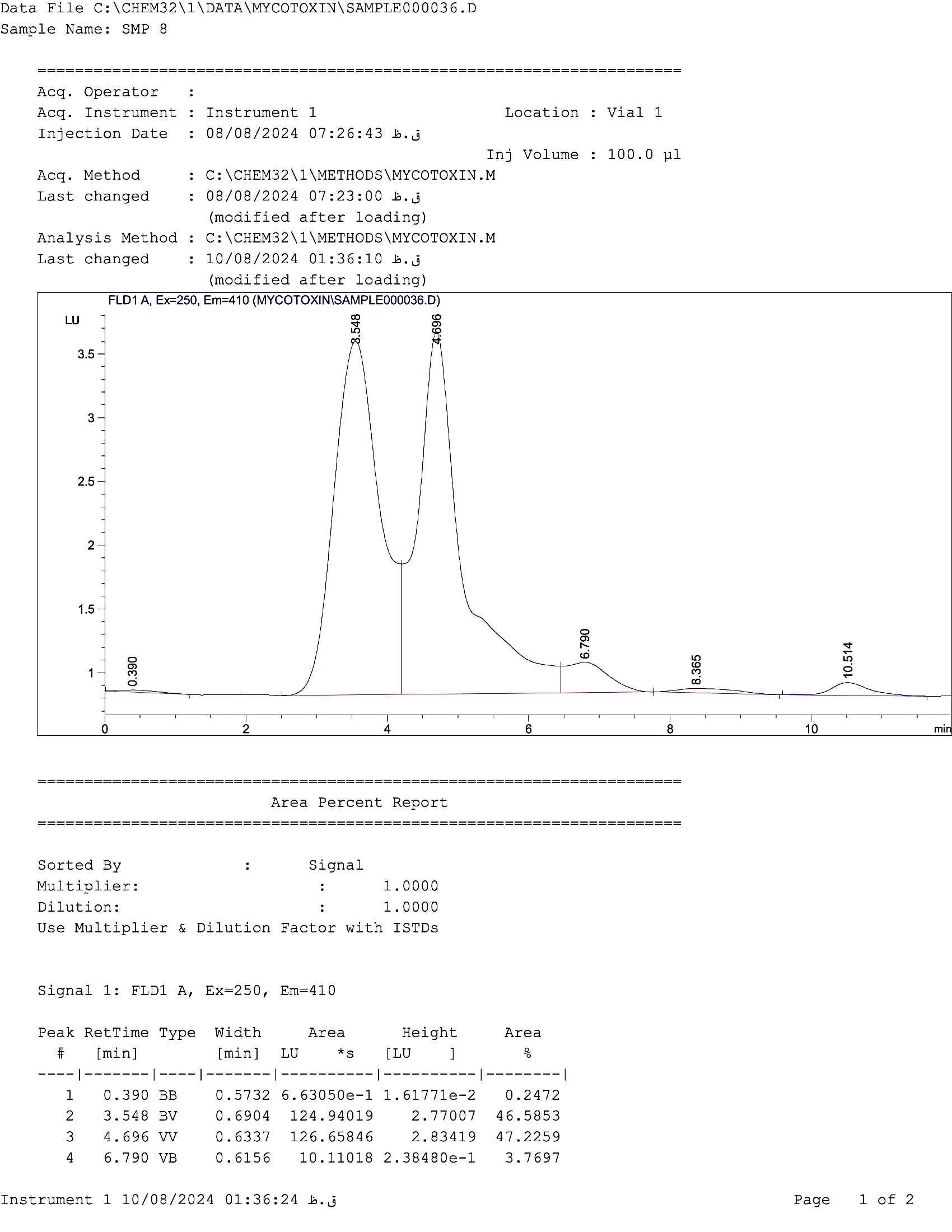


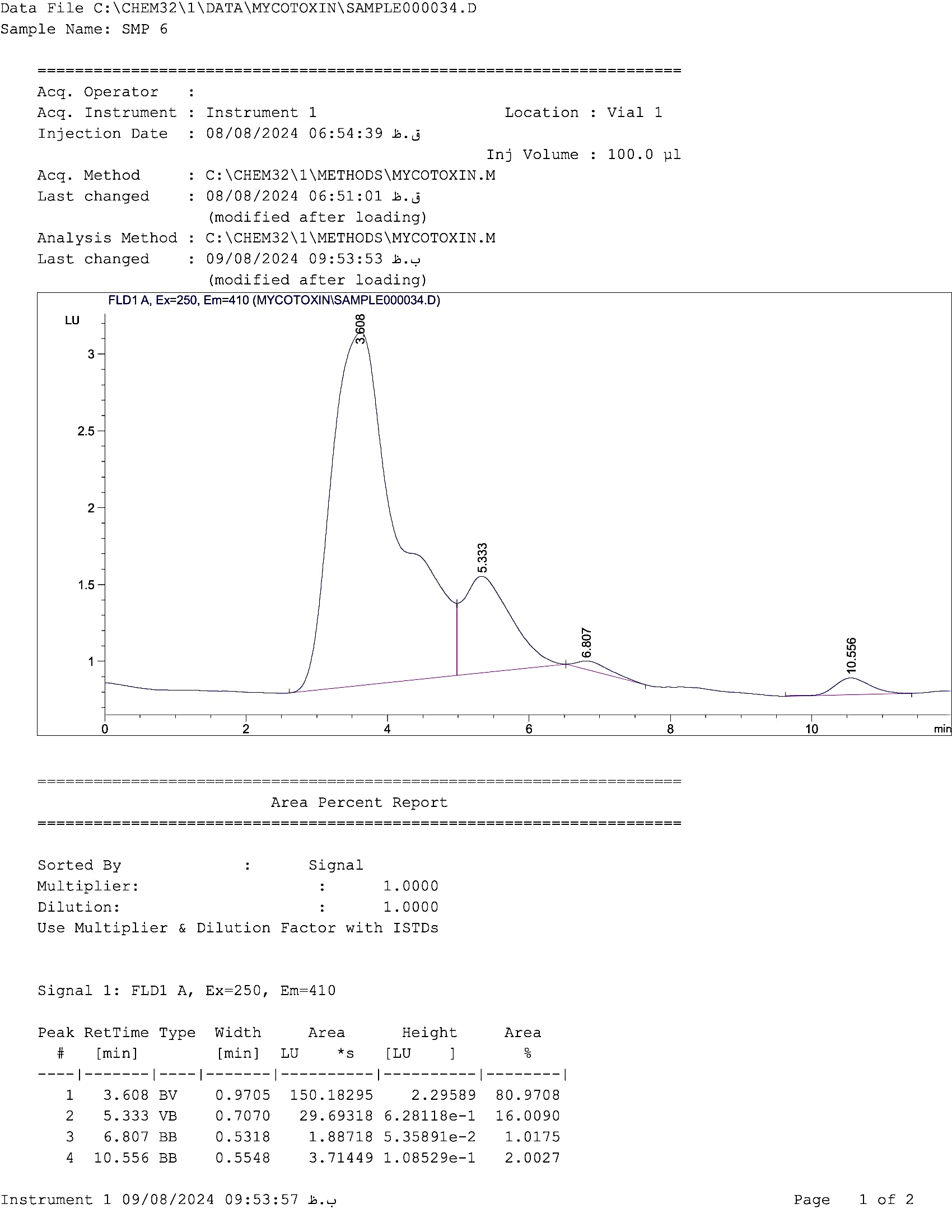


e


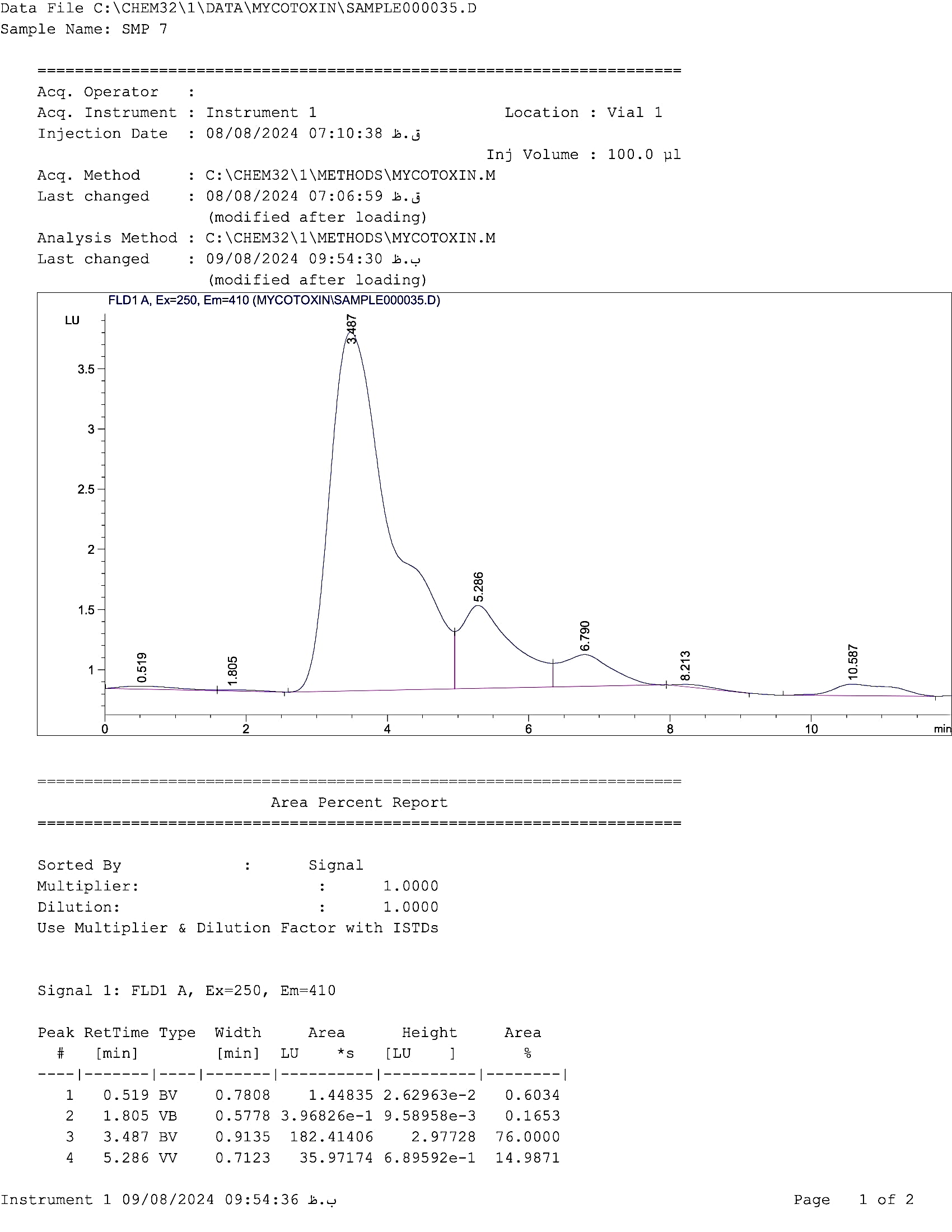


f


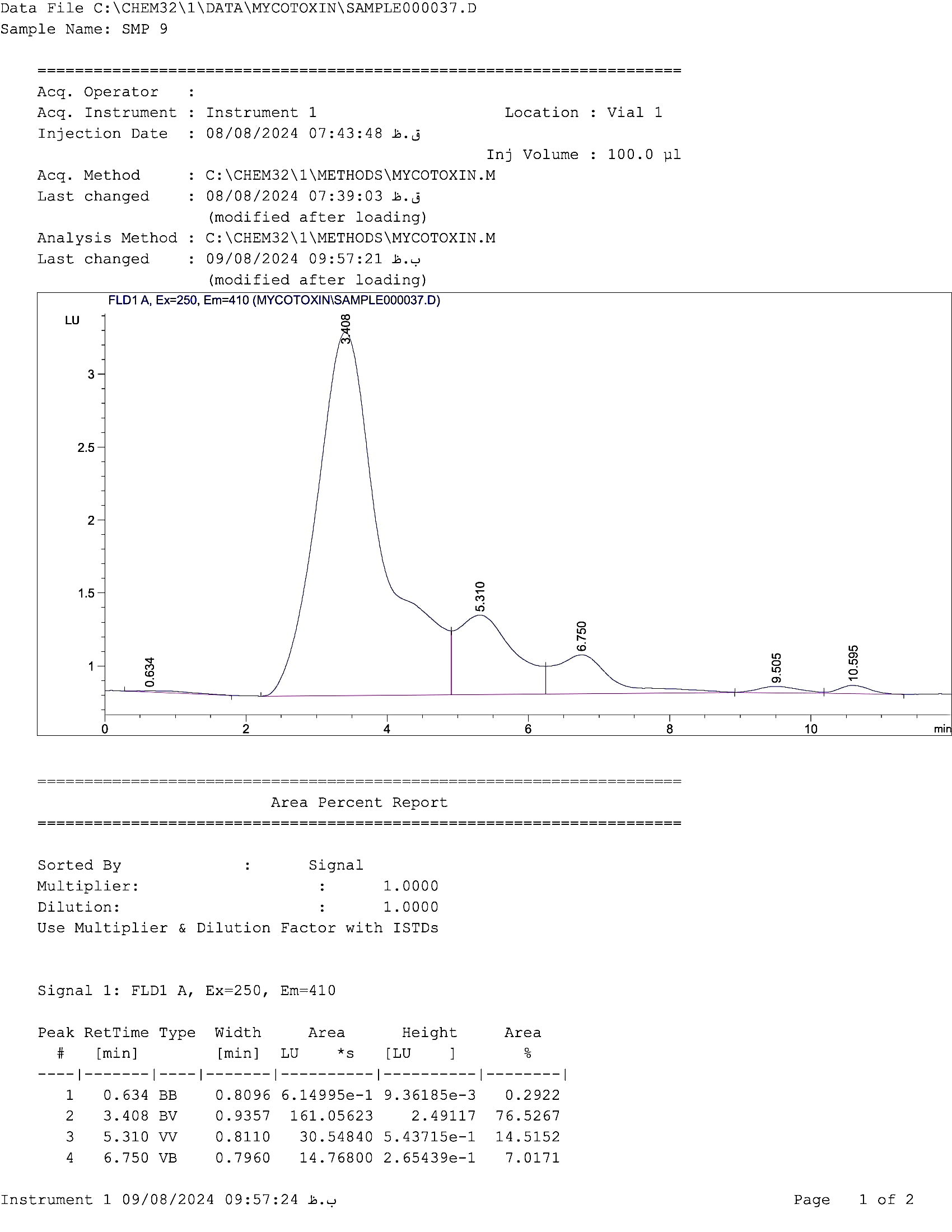


g

h


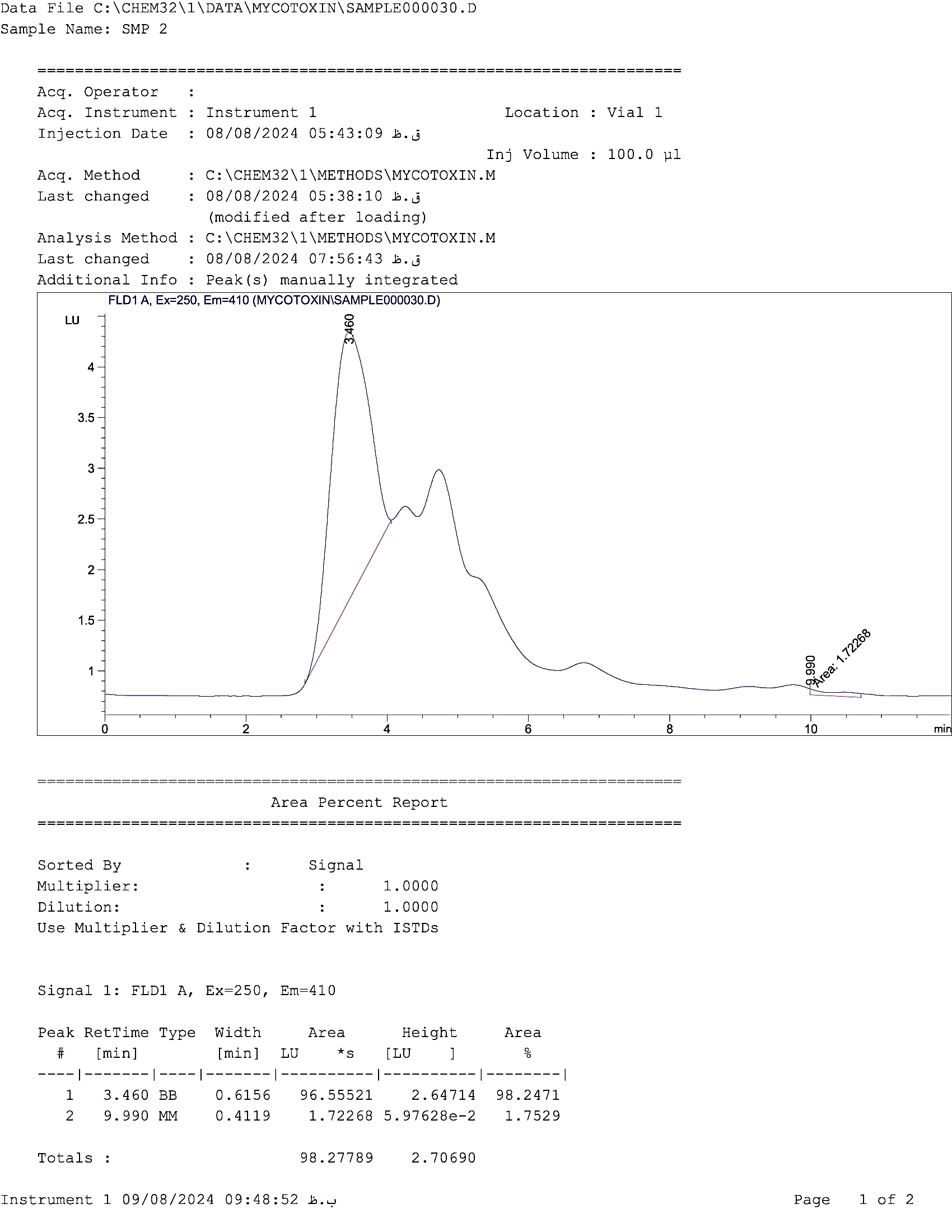


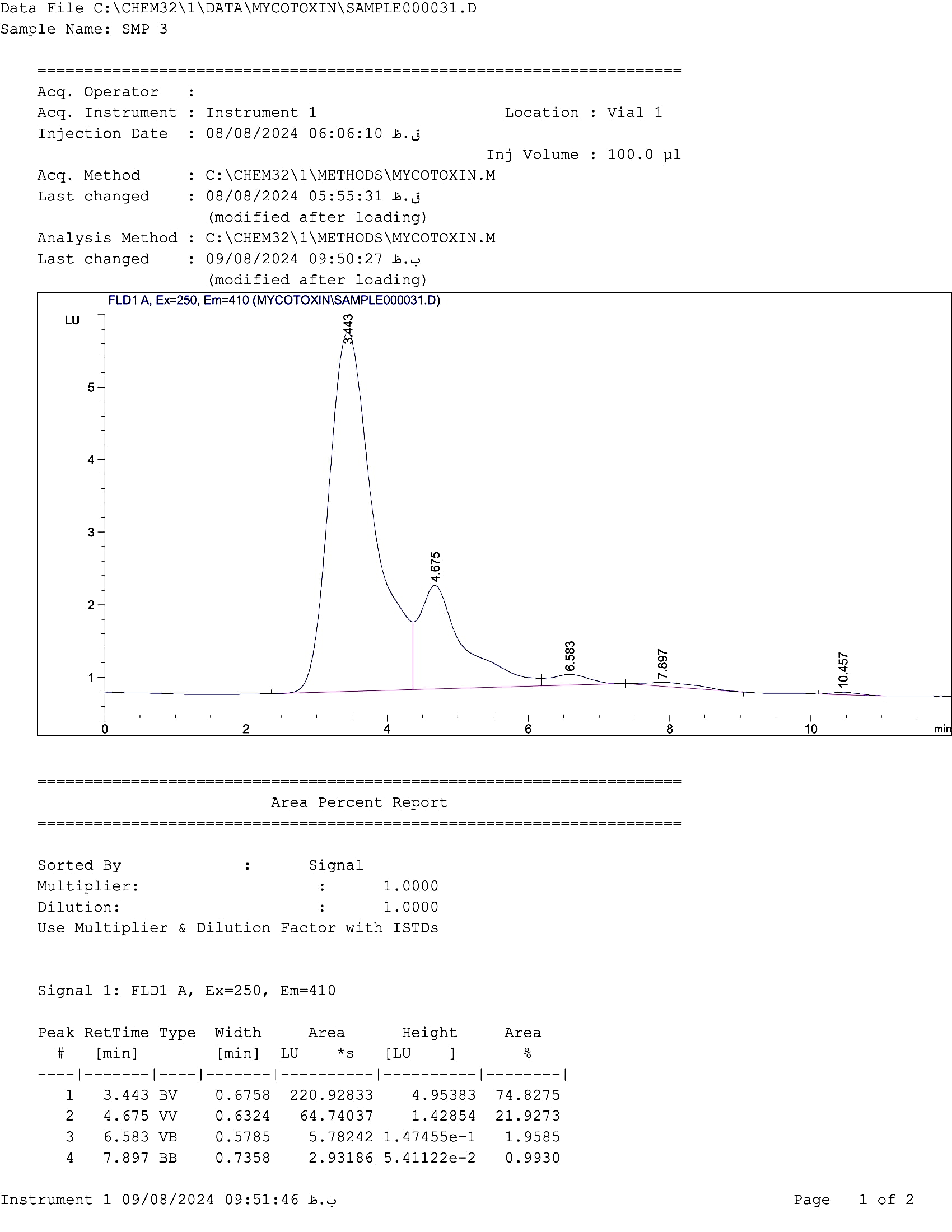


i

Figure 6s. HPLC chromatogram in PDM_LP_ (a); PDM_LRE_ (b); PDM_LR_(c); PDM_LP-LRE_ (d); PDM_LRE-LR_ (e); PDM_LR-LP_ (f); PDM_LRE-LR-LP_ (g); Yogurt (h); FDD-LA (i), PDM=Probiotic drink milk, FDD-LA=Fermented dairy drink containing Lactobacillus acidophilus, AFM1= Aflatoxin M1, LP=Lactobacillus plantarum, LRE=Lactobacillus reuteri, LR= Lactobacillus rhamnosus, LA= Lactobacillus acidophilus , PDM= Probiotic drink milk, PDM_LP_= PDM contains LP, PDM_LRE_=PDM contains LRE, PDM_LR_= PDM contains LR, PDM_LP-LRE_= PDM contains LP& LRE, PDM_LRE-LR_=PDM contains LRE & LR, PDM_LR-LP_=PDM contains LRE & LP, PDM_LRE-LR-LP_=PDM contains LRE, LR & LP, Yogurt= contains starter culture, FDD-LA=Fermented dairy drink containing Lactobacillus acidophilus
